# Multiple regions of E6AP (UBE3A) contribute to interaction with papillomavirus E6 proteins and the activation of ubiquitin ligase activity

**DOI:** 10.1371/journal.ppat.1008295

**Published:** 2020-01-23

**Authors:** Camille M. Drews, Nicole Brimer, Scott B. Vande Pol

**Affiliations:** Department of Pathology, University of Virginia, Charlottesville, Virginia, United States of America; The University of Manchester Faculty of Biology Medicine and Health, UNITED STATES

## Abstract

The HECT domain E3 ubiquitin ligase E6AP (UBE3A) is critical for the development of human papillomavirus (HPV) associated cancers, the neurodevelopment disorder Angelman Syndrome, and some cases of autism spectrum disorders. How E6AP recognizes its cellular targets and how its ubiquitin ligase activity is triggered remain poorly understood, and HPV E6 proteins are models for these processes. We examined diverse E6 proteins from human and non-human papillomaviruses and identified two different modes of interaction between E6 and E6AP. In Type I interactions, E6 can interact directly with the LXXLL peptide motif alone of E6AP (isolated from the rest of E6AP), and then recruit cellular substrates such as p53. In Type II interactions, E6 proteins require additional auxiliary regions of E6AP in either the amino terminus or in the carboxy-terminal HECT domain to interact with the LXXLL peptide motif of E6AP. A region of E6AP amino-terminal to the LXXLL peptide motif both augments association with E6 proteins and is required for E6 proteins to trigger ubiquitin ligase activity in the carboxy-terminal HECT ubiquitin ligase domain of E6AP. In Type I interactions, E6 can associate with E6AP and recruit p53, but a Type II interaction is required for the degradation of p53 or NHERF1. Interestingly, different E6 proteins varied in E6AP auxiliary regions that contributed to enhanced association, indicating evolutionary drift in the formation of Type II interactions. This classification of E6-E6AP interaction types and identification of a region in the E6AP amino terminus that is important for both E6 association and stimulation of ubiquitin ligase activity will inform future structural data of the E6-E6AP complex and future studies aiming to interfere with the activity of the E6-E6AP complex.

## Author summary

The cellular HECT domain E3 ubiquitin ligase E6AP (UBE3A) associates with papillomavirus E6 oncoproteins, which contribute to 5% of cancer deaths world-wide. In addition, loss of E6AP causes the neurodevelopmental disorder Angelman Syndrome, and overexpression of E6AP causes some cases of autism spectrum disorders. How E6AP recognizes its cellular targets, and how E6AP ubiquitin ligase activity is triggered remain poorly understood. The interaction of HPV E6 proteins with E6AP are models for these processes as E6 both recruits substrates to E6AP and activates its ubiquitin ligase activity. This study utilizes a diverse panel of E6 proteins and E6AP mutants to identify regions of E6AP that contribute to both the interaction of E6 with E6AP and the activation of ubiquitin ligase activity by E6.

## Introduction

Papillomaviruses induce epithelial hyperplasia (papillomas) in which the virus replicates under the control of virus-encoded E1 and E2 proteins [1, 2]. The virally encoded E5, E6, and E7 oncoproteins contribute to the formation of the papilloma, and are expressed under the transcriptional control of cellular transcription factors together with the E1 and E2 proteins [3–10]. All papillomaviruses encode oncoproteins, but certain particular papillomavirus types may not encode all three oncoproteins (E5, E6, or E7). The complete papillomavirus replication cycle can be studied using keratinocyte organotypic culture and cloned viral DNA [11, 12].

The E6 and E7 oncoproteins hijack the activities of cellular proteins, in particular certain E3 ubiquitin ligases, and alter the protein landscape of the cell by targeting cellular proteins for degradation [13–16]. Prolonged expression of the viral E6 and E7 oncoproteins by particular types of papillomaviruses (deemed high-risk) can lead to oncogenic transformation of the originally benign papilloma, while other papillomavirus types (deemed low-risk) are responsible for cutaneous warts, genital warts, and oropharyngeal papillomas that rarely progress to cancer but can be medically serious due to the size and location of the papilloma [17, 18]. The prototypic high-risk HPV types HPV16 and HPV18 are responsible for over 66% of cervical cancer cases in the United States [19]; HPV6 and HPV11 are prototypic low-risk types.

Papillomavirus E6 oncoproteins interact with target cellular proteins through docking on short sequences containing an LXXLL motif [20–22], and complexes of E6 + LXXLL peptides have been crystalized [23]. Characterization of E6 proteins from both human and non-human papillomaviruses has identified two broad but distinct groups with two differing functions. In the first E6 group, E6 proteins bind an LQELL motif on the cellular E3 ubiquitin ligase E6-associated protein (E6AP) to stimulate its ubiquitin ligase activity. E6AP is the product of the UBE3A gene and is regulated in neurons by genetic imprinting in the brain. Loss of E6AP expression leads to the development of Angelman syndrome [24, 25], while copy number variations (CNVs) resulting in overexpression of E6AP result in the development of autism spectrum disorders (ASDs) [26]. The second broad group of E6 proteins bind an LDDLL or LDALL motif on either the MAML family of transcriptional co-activators and/or paxillin to repress activation of Notch signaling [27–31]. E6 proteins with these two different cellular binding targets phylogenetically cluster separately in evolution, and can group most known papillomaviruses into two large supergenera: the E6AP-directed E6 alpha-supergenera and the MAML-directed E6 beta-gamma-supergenera [27]. Despite this evolutionary divergence, the overall protein fold of E6 complexed with their respective E6AP or paxillin-derived LXXLL peptides is quite similar [23, 32].

High-risk HPV E6 interacts with E6AP and then recruits p53, thereby triggering the ubiquitination of p53 and its subsequent proteasome-mediated degradation; neither E6 nor E6AP interact with p53 alone [33–37]. The ubiquitin ligase activity of E6AP can be ablated by mutation C843A (numbering from E6AP isoform II) in the ubiquitin binding site of E6AP; this mutant binds E6 and recruits p53, yet fails to ubiquitinate or degrade p53 [38]. The ability of high-risk HPV16 E6 (16E6) to interact with p53 is conferred when E6 binds to the isolated 12 amino acid LQELL peptide of E6AP, demonstrating that the specificity for interaction with p53 resides within the E6 protein [39]. The crystal structure of the 16E6 + LQELL peptide + p53 complex has been determined [40].

Most studies of E6 proteins have focused upon high-risk E6 and its substrate p53, but we recently identified NHERF1 as a cellular degradation target that is common to both high and low-risk E6 proteins, as well as E6 proteins from non-human papillomaviruses that associate with E6AP; the targeted degradation of NHERF1 by high and low-risk E6 proteins results in the activation of canonical Wnt signaling [41].

Knocking down E6AP in human papillomavirus-transformed cells rescues p53 expression and can lead to p53-induced apoptosis [42, 43]. Therefore, characterizing the interaction between E6AP and E6 is of great medical importance. Huibregtse et al. identified functional domains of E6AP required for *in vitro* 16E6 binding, p53 recruitment, and the targeted degradation of p53 by the 16E6-E6AP complex [37]. They demonstrated that everything carboxy-terminal to E6AP residue 290 was required for E6-dependent ubiquitination and the same region shortened on the carboxy terminus by only 84 amino acids was required for p53 association in the presence of 16E6. The same study identified an 18 amino acid peptide within the E6AP protein (containing the LXXLL motif) as both necessary and sufficient for 16E6 binding to E6AP.

Little is known about how (or if) E6 interacts with other regions of E6AP beyond the LQELL-containing peptide. But several observations indicate that the E6-E6AP interaction is more consequential than E6 simply docking upon the LQELL peptide alone. First, low-risk HPV11 E6 (11E6) does not detectably interact with the isolated LQELL peptide, while interacting robustly with that peptide when expressed in the context of full-length E6AP [39, 44, 45]. Second, when the LQELL peptide from E6AP is mutated to LQELS, 16E6 cannot bind the isolated E6AP LQELS-mutated peptide, but interacts robustly with LQELS in the context of full-length E6AP and can initiate p53 degradation [44]. Like the degradation of p53 by 16E6, 11E6 (which fails to bind the isolated E6AP LQELL peptide) can direct the degradation of NHERF1 in an E6AP-dependent manner [41]. Conversely, when the E6AP LQELL motif is mutated to reflect the LXXLL motif found in MAML1 (LDDLL), E6 proteins that bind MAML1 and not E6AP (such as E6 proteins from beta or gamma genera HPV) display a gain-of-function binding to the E6AP-LDDLL chimera; although these MAML1-binding E6 proteins can now bind full-length E6AP-LDDLL, they are unable to stimulate E6AP ubiquitin ligase activity [27]. Therefore, an E6 protein binding at the E6AP LXXLL motif is not sufficient *per se* to initiate E6AP ubiquitin ligase activity. We hypothesize that, beyond the HECT ubiquitin ligase domain, there are additional regions in E6AP located amino-terminal to the LQELL motif, that are necessary for E6 activation of E6AP ubiquitin ligase activity.

In this study, we characterize regions in both the amino and carboxy-termini of E6AP that are important in mediating the E6-E6AP interaction, in E6 stimulation of E6AP ubiquitin ligase activity, and in targeted degradation of p53 and NHERF1 by both high and low-risk E6 proteins. We find that E6AP-associating E6 proteins isolated from human and non-human hosts require different regions within E6AP to successfully form the E6-E6AP complex. Our observations have led us to classify E6 interactions with E6AP into two distinct types. In Type I interactions, E6 proteins have a direct interaction with the isolated E6AP LQELL peptide whereas in Type II interactions, E6 proteins have a facilitated interaction with the LQELL motif in the context of the full-length E6AP protein. We identified a mutant of high-risk 16E6 that is incapable of binding the isolated E6AP LQELL motif in a Type I interaction, but retains its ability to interact with the full-length E6AP protein in a Type II interaction. Using this 16E6 mutant, we identified regions in E6AP outside the LQELL binding-peptide that enhance 16E6 interaction with E6AP in the amino-terminus, the central portion of E6AP, and in the carboxy-terminal HECT domain. We found that a given E6 protein may or may not display a Type I interaction with E6AP, but to target degradation of a substrate, E6 proteins require a Type II interaction. Finally, we find that although the E6-E6AP interaction is conserved among E6 proteins isolated from different vertebrate species, these E6 proteins subtly differ in their interactions with E6AP.

## Results

### Diverse papillomavirus E6 proteins preferentially bind full-length E6AP compared to the isolated E6AP LQELL motif

**Fig 1:**
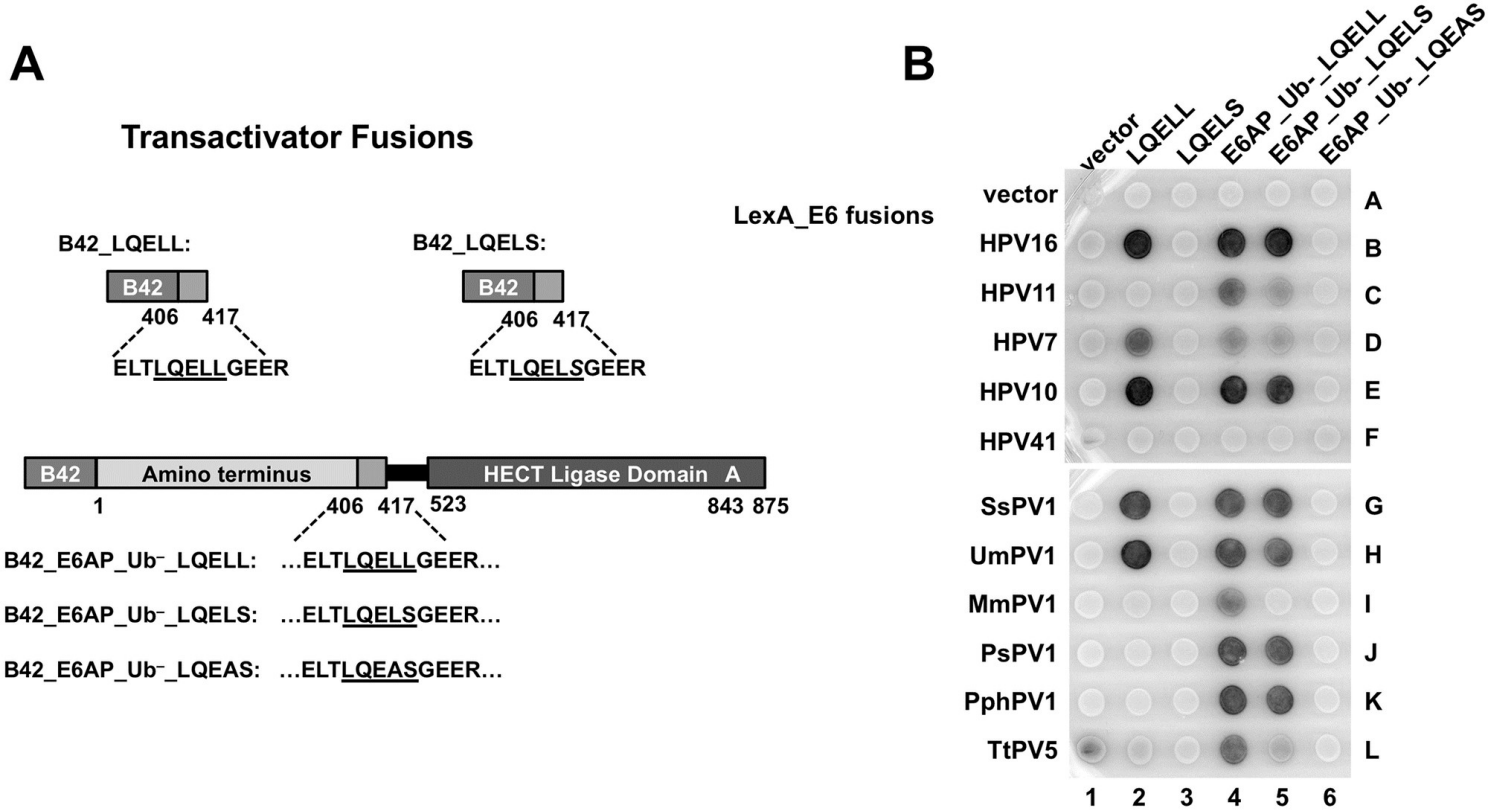
E6 proteins display different E6AP interaction profiles. (A) Schematic of B42 transactivator-domain E6AP fusion proteins utilized in panel B. B42_LQELL contains E6AP residues 406–417. B42_LQELS also contains E6AP residues 406–417 but the double LL (residues 412 and 413) have been mutated to LS. B42_E6AP_Ub^–^_LQELL consists of ubiquitin ligase dead full-length E6AP. B42_E6AP_Ub^–^_LQELS is also ubiquitin ligase dead full-length E6AP, but is mutated in the LQELL motif to LQELS, and similarly B42_E6A_Ub-LQEAS as indicated. **(B)** E6 proteins from human and animal papillomaviruses have different requirements for interaction with E6AP. Bait yeast strains expressing the LexA DNA binding domain fused to E6 proteins from the listed papillomaviruses were mated to prey yeast expressing B42 transcriptional activation domain fusions illustrated in part A. HPV41 E6 preferentially binds MAML1 and not E6AP, and was used as a negative control for binding. The horizontal white line indicates development on parallel matched XGAL plates. H = *Homo sapiens* (human), Ps = *Phocoena spinipinnis* (Burmeister’s porpoise), Pph = *Phocoena phocoena* (harbor porpoise), Tt = *Tursiops truncatus* (bottlenose dolphin), Mm = *Macaca mulata* (rhesus monkey), Ss = *Sus scrofa* (wild boar), Um = *Ursus maritimus* (polar bear).

High-risk 16E6 binds the isolated E6AP LQELL peptide [39], while interaction of the low-risk 11E6 is not detected [45]. To determine how other diverse E6 proteins interact with E6AP, we first examined a cohort of E6 proteins that all interact with E6AP from human (HPV16, 11, 7, and 10), cetacean (PsPV1, PphPV1, TtPV5), primate (MmPV1), and ungulate (SsPV1 and UmPV1) hosts for their ability to interact in vivo with full-length E6AP compared to the isolated E6AP LQELL peptide. We made full-length E6AP with either the wildtype (WT) E6 binding site LQELL, or singly mutated LQELS or doubly mutated LQEAS docking sites, as well as transactivator fusions to the LQELL peptide alone or a transactivator fusion to the mutated LQELS peptide (Fig 1A). The full-length E6AP constructs were ubiquitin ligase inactive (designated as Ub^–^), due to mutation of the active site cysteine residue to an alanine (C843A). We expressed the E6 proteins as LexA fusions and the various E6AP proteins as transactivator fusions in a yeast two-hybrid system to detect the formation of the heterodimeric complex *in vivo* as a dark XGAL yeast patch (Fig 1B). HPV41 E6 (41E6) interacts with MAML1 and not E6AP [27], and was used as a negative control (Fig 1B, row F).

All of the E6 proteins interacted with full-length E6AP_Ub^–^ as expected. About half of the E6 proteins (HPV16, HPV7, HPV10, SsPV1, and UmPV1) interacted with the isolated E6AP LQELL peptide (Fig 1B, spots 2B, 2D, 2E, 2G, 2H) while the other half did not detectably interact with the isolated LQELL peptide (HPV11, PsPV1, PphPV1, TtPV5, or MmPV1). Although none of the observed E6 proteins bound the isolated, mutated LQELS peptide (Fig 1B, column 3), many interacted with LQELS within the context of full-length E6AP_Ub^–^(with the exception of TtPV5 E6 and MmPV1 E6, Fig 1B, spot 4I vs. 5I and spot 4L vs. 5L). But further mutation of the LQELL motif in E6AP_UB- from LQELS to LQEAS ablated detectable interaction of any E6 protein with E6AP_Ub-. We concluded that numerous E6 proteins (that did not detectably interact with the isolated LQELS but could interact with LQELS in the context of full-length E6AP), were similar in this regard to HPV11 E6. This observation led us to hypothesize that other regions within E6AP, in addition to the alpha-helical LQELL motif, contribute to the E6-E6AP interaction. We term the interaction of an E6 protein with the isolated LQELL motif a Type I interaction, and an interaction that requires additional domains in E6AP a Type II interaction.

### The 16E6_L50A mutation enables the identification of additional E6AP regions that contribute to the 16E6-E6AP association

We wished to gain further insight into E6 proteins that interact with E6AP but fail to interact with the isolated LXXLL motif, such as 11E6, and the additional E6 types shown in Fig 1B. However, it would be useful to study this phenomenon using an E6 type where the structure has been determined. As yet, only the 16E6 and BPV1 E6 structures have been determined, and of these two, only 16E6 associates with E6AP and it requires just the E6AP LQELL motif for interaction [23]. We hypothesized that a 16E6 mutant could be impaired in its association with the isolated LQELL peptide, yet retain its interaction with LQELL in the context of the full-length E6AP, and thus resemble 11E6 and other E6 proteins such as those from PsPV1, PphPV1, TtPV5, and MmPV1.

**Fig 2:**
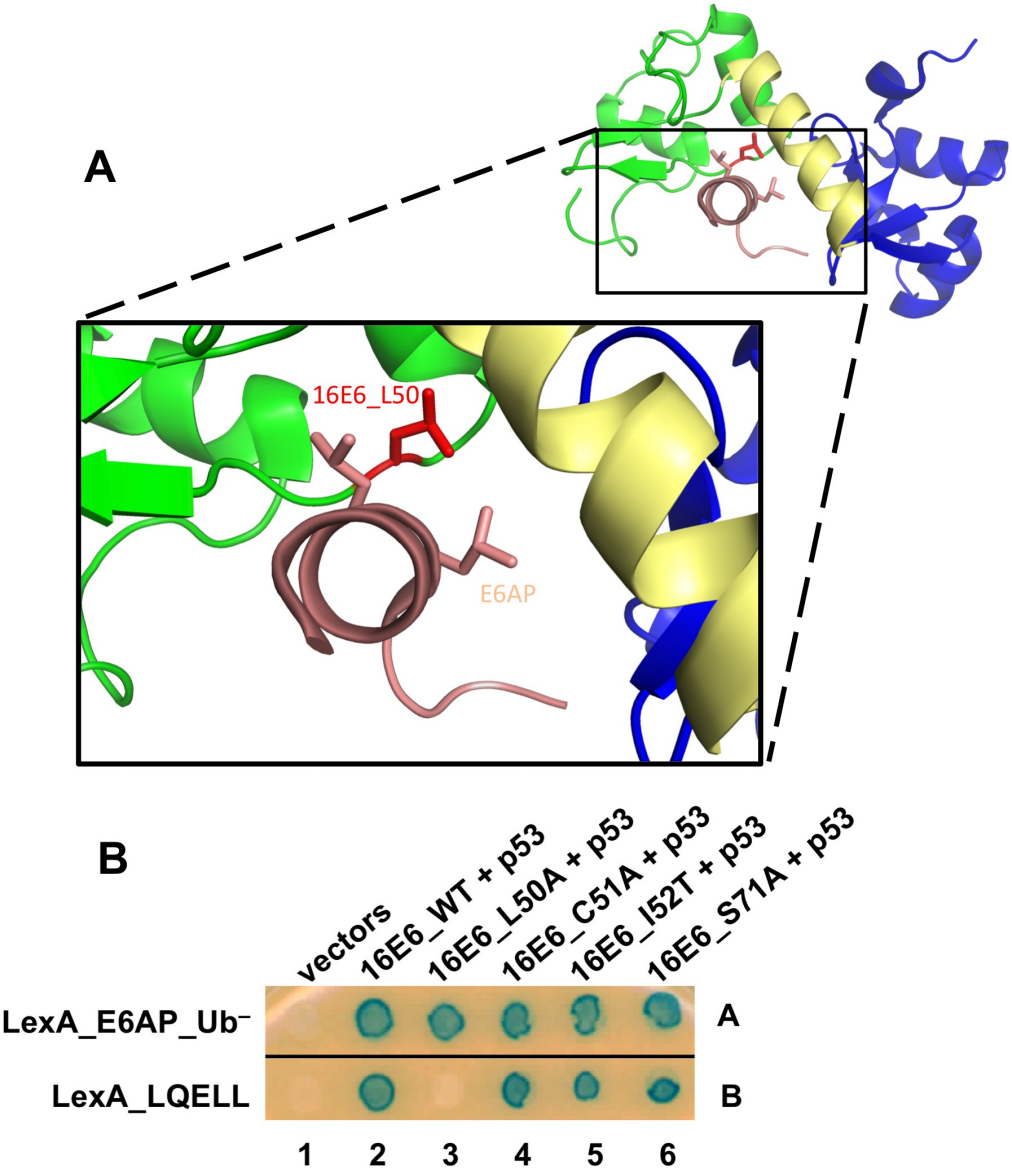
The 16E6_L50A mutant enables characterization of E6-E6AP interactions. **(A)** The E6 L50 residue is in close proximity to the double L residues in the E6AP LQELL peptide. The HPV16 E6 structure (PDB file 4GIZ) is depicted with the amino terminal zinc-structured domain in green, the carboxy terminal zinc-structured domain in blue, and the connecting alpha helix in yellow. The 16E6 protein is shown interacting with the E6AP LQELL peptide (in light pink), and the side chains of the double L residues are shown. The side chain of the E6 L50 residue is highlighted in red. The E6 L50 residue (red) makes a 3.7 Å side chain contact with the last leucine residue (L) in the E6AP LQELL peptide which is lost upon mutation of L50 to an alanine. **(B)** Mutation of 16E6 L50 residue to an alanine results in a high-risk E6 protein that is unable to bind the isolated E6AP LQELL peptide but retains association with full-length E6AP. Bait Yeast expressing LexA fused to either full length ubiquitin ligase dead E6AP (E6AP_Ub^–^) or the isolated E6AP LQELL peptide (E6AP_406–417) were mated to yeast co-expressing untagged 16E6_WT or the indicated 16E6 mutants containing a single amino acid change and p53.

To test this hypothesis, we created a structure-informed mutant of 16E6 (16E6_L50A) to impair binding of 16E6 to the isolated E6AP LQELL peptide [23]. In Fig 2A, the 16E6 L50 residue (red) makes a 3.7 Å contact with L413 found in the E6AP LQELL peptide (Fig 2A, shown in light pink [23]). We utilized a yeast-three hybrid (Y3H) system [39] in which 16E6 is expressed in its native form. This system enables observation of trimeric complex formation, where E6AP is fused to LexA, E6 is expressed in its native state, and the remaining associating protein has transactivator activity (either via a fusion or intrinsically). In this instance, 16E6 recruits p53 to LexA_E6AP_Ub^–^ or LexA_E6AP LQELL peptide alone (Fig 2B). While wildtype 16E6 could recruit p53 to either E6AP_Ub- or to the isolated LQELL peptide (as we previously observed [39]), 16E6_L50A could only recruit p53 to the full-length E6AP and failed to recruit p53 to the isolated E6AP LQELL peptide (Fig 2B, compare spots 3A and 3B). 16E6_R102 also makes close contact with E6AP L413, and has a similar interaction profile as 16E6_L50A (S1 Fig).

### E6AP from aa. 300–403 confers both enhanced association of E6 with E6AP and is required for ubiquitination of p53

**Fig 3:**
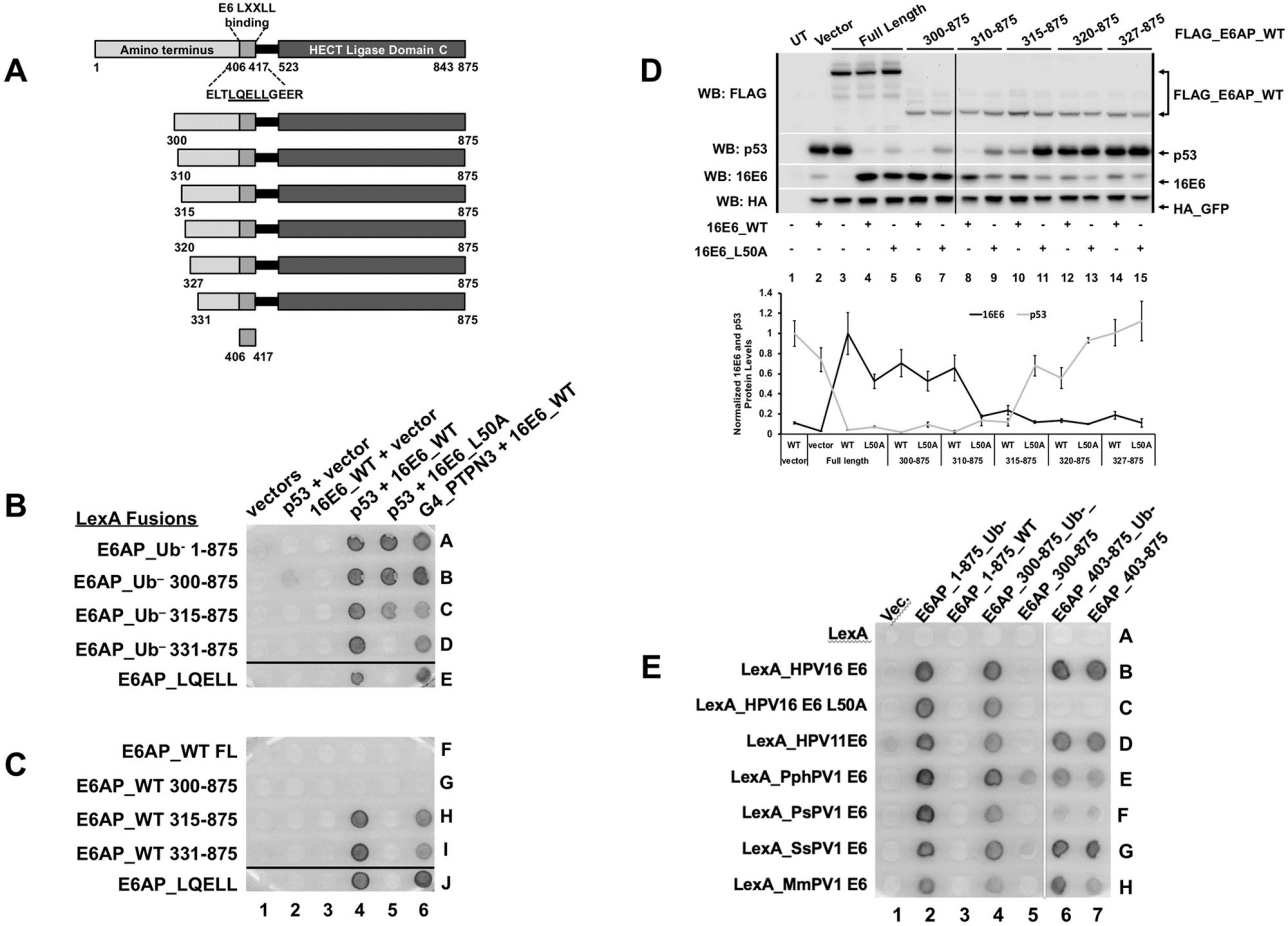
The amino terminal region of E6AP is required for 16E6 degradation of p53. **(A)** Schematic of E6AP amino terminal truncations. Previously described E6 LQELL binding region is between amino acids 403 and 416, as depicted. Each E6AP amino terminal truncation retains the E6 LQELL binding region and the E6AP carboxy terminal HECT ligase domain. (**B)** Ability of 16E6_L50A to bind to E6AP requires E6AP residues 315–331. Yeast strains expressing the LexA DNA binding domain fused to ubiquitin ligase dead (Ub–) E6AP full length (FL), amino terminal E6AP truncations, or the isolated E6AP LQELL peptide (406–417). These bait yeasts were mated to yeast strains co-expressing p53, 16E6_WT, 16E6_L50A, or transactivator Gal4 (G4) fused to the PDZ protein PTPN3 as indicated. Positive controls include 16E6_WT co-expressed with p53 to ensure E6AP expression and G4-PTPN3 co-expressed with 16E6_WT to ensure 16E6_WT expression. Ability of 16E6_L50A to bind E6AP_Ub- and recruit p53 is lost when E6AP is truncated from residue 315 to residue 331. Horizontal black line indicates removal of an irrelevant sample. **(C)** Ability of E6 to stimulate ubiquitin ligase activity in the complex with p53 and PTPN3 requires E6AP amino acids 310–315. Bait yeast were transfected with the LexA DNA binding domain fused to WT E6AP containing the same truncation endpoints as described in B. These yeast strains were mated to the same yeast as described in B and diploids selected. Upon truncation of E6AP from amino acid 300 to 315, the interaction of p53 and PTPN3 with E6 was restored as indicated by the appearance of blue dye. Horizontal black line indicates removal of irrelevant samples. **(D)** Requirement of E6AP amino acids 310–320 for E6 ability to initiate p53 degradation in the presence of E6AP_WT is recapitulated in E6AP-null 8B9 cells. Plasmids encoding the indicated FLAG_E6AP_WT truncations (1.25 ug), human p53 (0.5 ug), HA-GFP (0.01 ug), and either untagged 16E6_WT or 16E6_L50A (2 ug), as indicated, were transiently transfected into murine E6AP-null 8B9 cells and p53 and E6 expression were analyzed by western blot. 16E6_WT requires E6AP amino acids 315–320 to initiate E6AP-mediated degradation of p53 while 16E6_L50A requires E6AP amino acids 310–315. A single representative blot is shown. Vertical black line indicates removal of an irrelevant sample. UT = untransfected. Quantitation of protein expression from triplicate experiments as shown below the blots. E6AP stabilization of 16E6 (black line) is not required for p53 (gray line) degradation by the E6-E6AP complex. p53 levels and E6 levels are normalized to co-transfected HA_GFP. HA_GFP normalized E6 expression levels are further normalized to 16E6_WT protein levels in the presence of full length E6AP (lane 4 in panel D). HA_GFP normalized p53 protein expression levels are normalized to p53 levels in the presence of 16E6_WT with no co-expressed E6AP protein (lane 2 in panel D). The means of triplicate independent experiments ± standard errors are shown. **E**. Diverse E6 proteins require E6AP region 300–403 for ubiquitin ligase function. The indicated LexA_E6 fusions from Fig 1 are expressed together with transactivator fusions of E6AP active for Ubiquitin ligase activity (WT) or Ubiquitin minus (Ub-) in columns, either full length E6AP_1–875, or amino-terminal truncations 300–875 or 403–875. White vertical line indicates the division from 2 matched plates.

Fig 2B showed that additional sequences in E6AP outside of the LXXLL motif contributed to the association of E6 with E6AP. We hypothesized that E6AP region(s) amino-terminal to the LQELL motif would be required for 16E6 ubiquitination of p53 *in vivo*. We first tested the E6-E6AP interaction in yeast by creating LexA-fused amino-terminal truncations of E6AP (Fig 3A) and employing a yeast 3-hybrid (Y3H) assay (Fig 3B). Interaction of 16E6_L50A with E6AP_Ub^–^ required the E6AP amino-terminal region between residues 315 and 331 (Fig 3B, compare spots 5C and 5D). Thus, 16E6_L50A, (which cannot associate with the isolated LQELL peptide but does associate with LQELL in the context of E6AP_Ub^–^ (Fig 2B)), requires E6AP sequences contained within amino acids 315–331 to associate with E6AP_Ub-. In yeast, E6AP_Ub^–^ recruits 16E6_WT and p53 resulting in a dark colony, while E6AP_WT (containing ubiquitin ligase activity) does not generate a dark colony in the presence of 16E6_WT and p53. Fig 3C shows that the ability of 16E6_WT to recruit p53 and activate the LexA responsive reporter was lost in the presence of E6AP_WT, but was restored upon truncation of E6AP_WT to amino acid 315, as indicated by the conversion of white colonies to dark colonies (Fig 3C, spot 4G compared to 4H). PTPN3 is included in Fig 3C as an additional positive control for the interaction, as it does not require E6 to recruit p53. Together, these data suggested that the amino terminal region of E6AP between amino acid 300 and 331 was involved in mediating both the enhanced interaction with E6AP and the ability of 16E6 to initiate ubiquitin ligase function.

To confirm in mammalian cells the results that were obtained in yeast, we performed transient transfections into murine E6AP-null 8B9 cells reconstituted with various amino-terminal truncations of ubiquitin-ligase active E6AP in the presence of either 16E6_WT or 16E6_L50A (Fig 3D). Both WT and L50A 16E6 targeted p53 for degradation in the presence of E6AP 310–875 (Fig 3D, p53 levels in lanes 8 and 9 vs lane 2). Upon further amino terminal truncation to residue 315, 16E6_WT still initiated p53 degradation, but 16E6_L50A did not (Fig 3D, compare p53 levels in lanes 10 and 11). 16E6-mediated p53 degradation was completely lost by both 16E6_WT and L50A after further truncating E6AP to create E6AP 320–875 (lanes 12 and 13).

We hypothesized that the ability of 16E6 to initiate degradation of p53 might be dependent upon its stabilization through E6AP interaction [46]. However, quantitation of p53 and 16E6 levels in the presence of the various E6AP truncation mutants examined in Fig 3D demonstrated that both 16E6_WT and 16E6_L50A were able to initiate p53 degradation when 16E6 protein levels were not obviously stabilized (Fig 3D). 16E6_L50A protein stabilization by E6AP was lost with E6AP truncation to 310–875 and 16E6_WT protein stabilization by E6AP was lost with E6AP truncation to 315–875, but in both cases p53 was still degraded (Fig 3D).

The results in Fig 3B, 3C and 3D show that activation of ubiquitin ligase function by16E6 requires the 300–403 region of E6AP. To determine if this region of E6AP was broadly required for ubiquitin ligase function in yeast by E6 proteins from different papillomavirus genera, an analysis of additional diverse E6 proteins showed a similar dependence upon amino acids 300–403 (Fig 3E).

**Fig 4:**
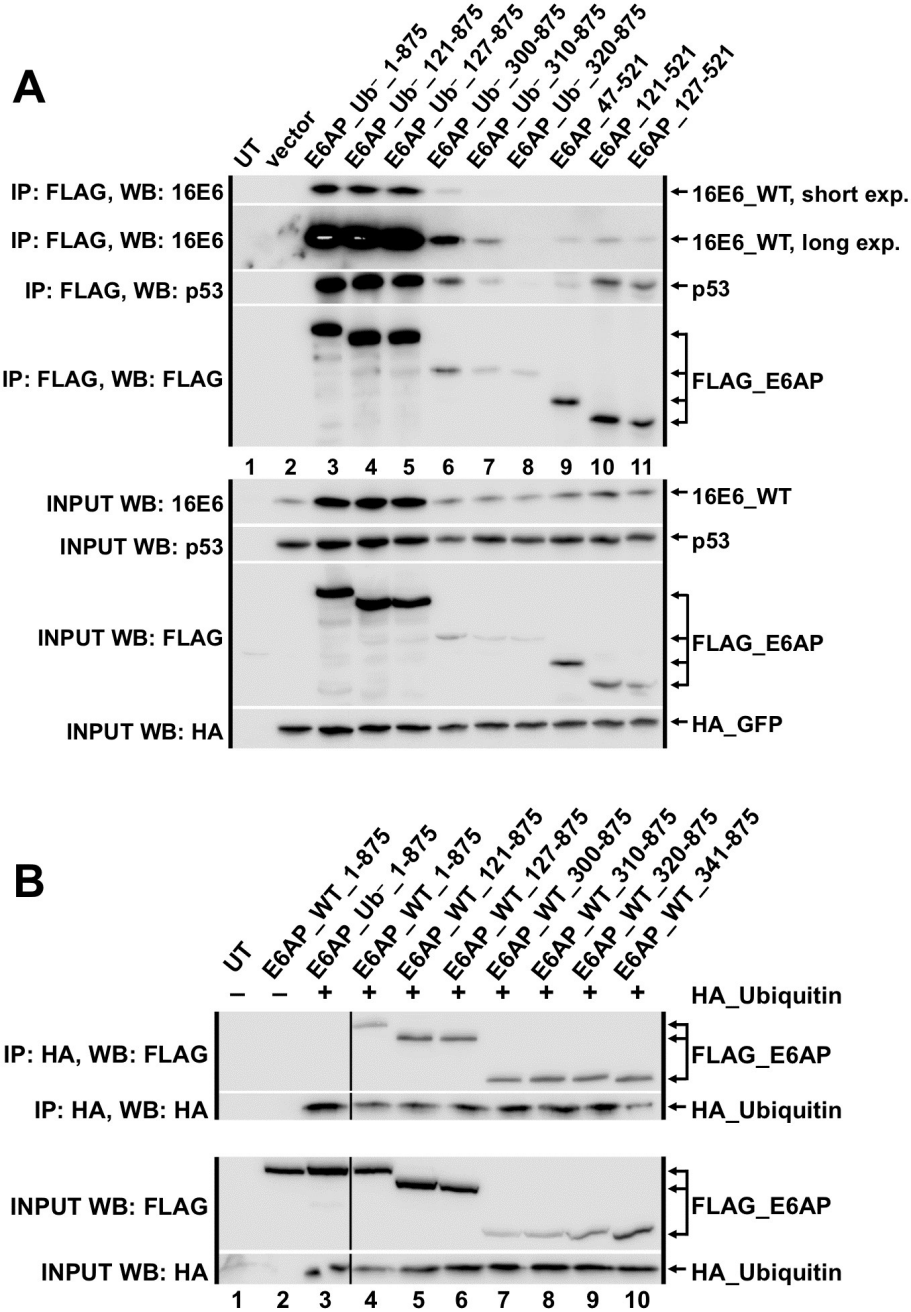
Co-immune precipitations between 16E6, fragments of E6AP, and human p53. **(A)** Untagged 16E6_WT (4 ug), human p53 (2.9 ug), HA_GFP (0.1 ug), and the indicated FLAG_E6AP_Ub^–^ truncations (3 ug) were co-transfected into E6AP-null 8B9 cells and harvested 18 hrs. later in 0.5X IGEPAL lysis buffer as described in methods. Western blots of input samples are clustered at the bottom and FLAG-immunoprecipitated (IP) samples are clustered at the top. Input was 5% of the immunoprecipitated sample size. The 16E6_WT IP blot shows a short and a long exposure; the overexposure is necessary to see 16E6_WT in lanes 7 and 9–11 (possibly due to lower expression of FLAG_E6AP truncations in those lanes). Ub^–^ indicates a ubiquitin ligase dead E6AP mutant. UT = untransfected. **(B)** Amino-terminal E6AP truncations maintain ability to be loaded with ubiquitin. HA_Ubiquitin (6 ug) and FLAG_E6AP (3 ug; either ubiquitin ligase dead (Ub^–^) or ubiquitin ligase active (WT)) plasmids were co-transfected into E6AP-null 8B9 cells and harvested 18 hrs. later in 0.5X IGEPAL lysis buffer as described in the methods. Western blots of input samples are clustered at the bottom and HA-immunoprecipitated samples are clustered at the top. Input was 5% of the immunoprecipitated sample size. E6AP_Ub^–^ indicates a ubiquitin ligase dead E6AP mutant. Black line indicated removal of an irrelevant sample.

To confirm the yeast results, tagged E6AP and fragments of E6AP together with E6 and p53 were co-expressed in E6AP null murine cells and subjected to immune precipitation. E6AP 320–875 was unable to coimmunoprecipitate 16E6_WT and p53 in E6AP-null 8B9 cells, while other less extensive E6AP truncations retained this ability (Fig 4A). We speculated that some of the E6AP truncations may be functionally dead in that their active cysteine residue could not be loaded with ubiquitin. However, this was not the case, as each E6AP truncation coimmunoprecipitated with ubiquitin (Fig 4B).

**Fig 5:**
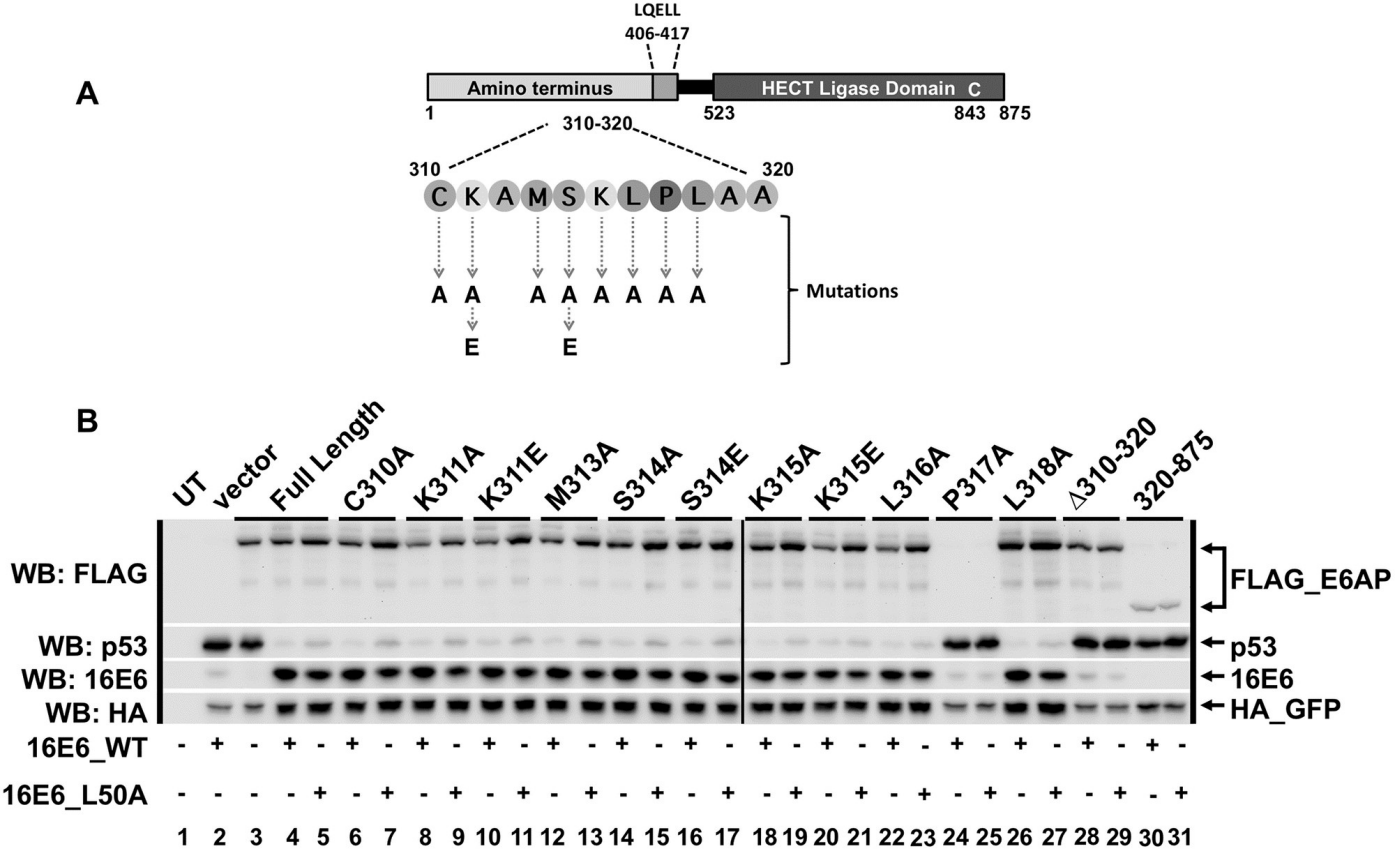
No single amino acid point mutation within E6AP region 310–320 prevents 16E6_WT from initiating degradation of p53. **(A)** Schematic of full length E6AP protein. The amino acids located between 310 and 320 (inclusive) are depicted, as is the location of the HECT ligase domain and the active cysteine residue (C843), responsible for ubiquitination of p53. **(B)** Full length E6AP containing single amino acid point mutations within the 310–320 region still degrade p53 in the presence of 16E6_WT. Plasmids encoding the indicated FLAG_E6AP (1.25 ug), human p53 (0.5 ug), HA_GFP (0.01 ug), and either 16E6_WT or 16E6_L50A (2 ug) were co-transfected into E6AP-null 8B9 cells. Δ310–320 is full length E6AP_WT deleted of residues 310–320 (inclusive). Amino terminal E6AP truncation 320–875 was used as a negative control for p53 degradation in the presence of either 16E6_WT or 16E6_L50A. Vertical black line indicates samples were run on two different western blots. UT = untransfected.

To identify whether specific residue(s) within E6AP region 310–320 were required for 16E6-mediated degradation of p53, we constructed single amino acid substitution mutants within residues 310–320 (inclusive) in the context of full-length E6AP (Fig 5A). These E6AP mutants were co-transfected with p53, HA_GFP, and either 16E6_WT or 16E6_L50A in E6AP-null 8B9 cells. None of the observed E6AP single amino acid point mutants prevented 16E6-mediated degradation of cellular p53 (Fig 5B). E6AP P317A was not expressed (lanes 24 and 25, likely because it conferred protein instability. We also made an internal deletion of residues 310–320 (inclusive) in the context of full-length E6AP (Δ310–320) and saw that this E6AP mutant, like the E6AP truncation mutant 320–875, resulted in loss of targeted p53 degradation by 16E6 (Fig 5B lanes 28–29). Secondary structure analysis predicts the 310–320 region to have alpha-helical structure. These mutational results suggest that maintenance of the alpha-helicity of this segment of the E6AP 310–320 region is important in enabling high-risk E6 degradation of p53.

### Low-risk and high-risk E6 proteins require the same amino-terminal region of E6AP to initiate degradation of cellular substrates

**Fig 6:**
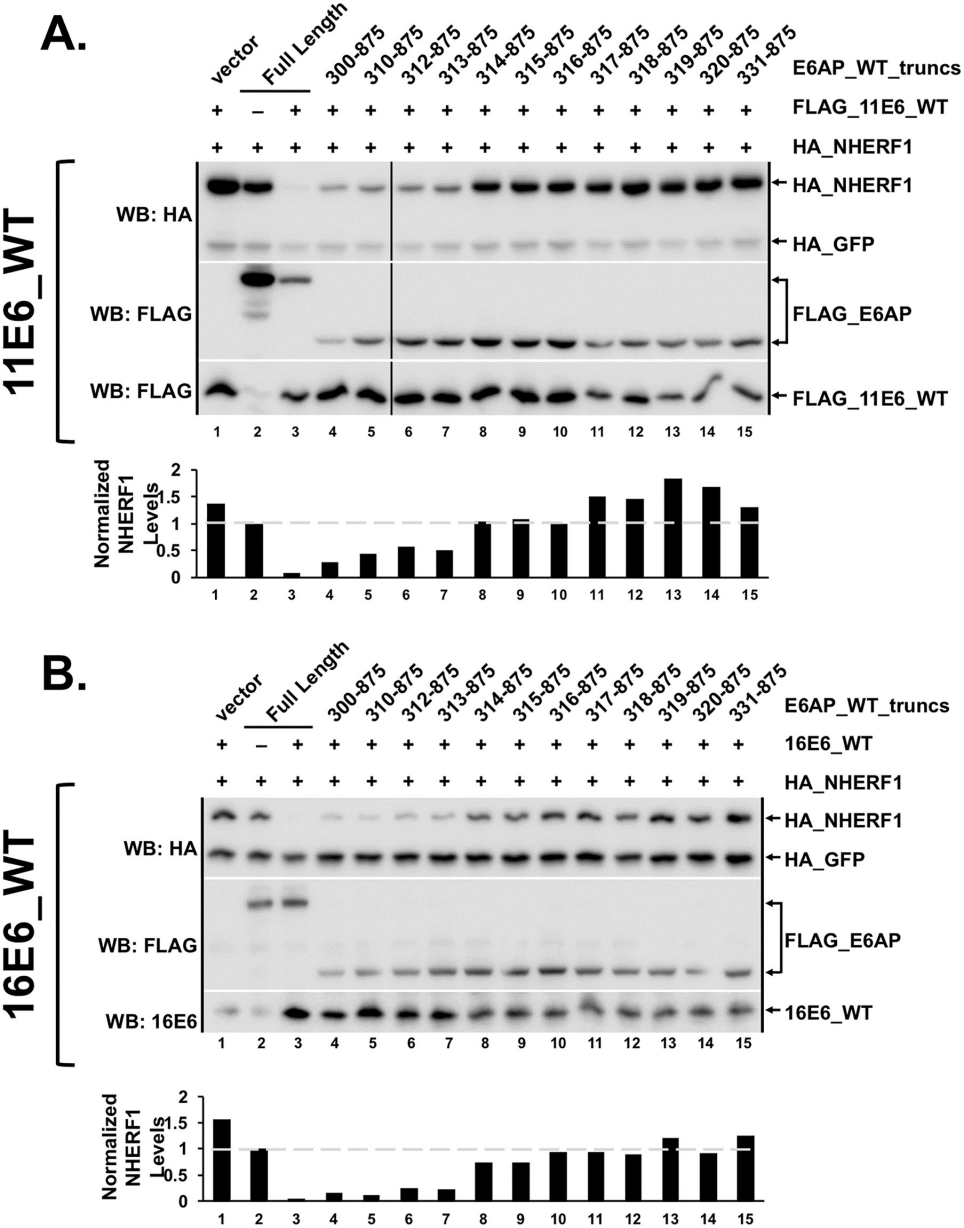
Low-risk and high-risk E6 proteins require the same amino terminal region of E6AP to initiate degradation of cellular substrates. NHERF1 degradation lost in the presence of a ubiquitin active truncation E6AP 314–875 with both **(A)** low-risk 11E6 and **(B)** high-risk 16E6. The listed FLAG_E6AP_WT truncations were co-transfected with HA_NHERF1 (0.75 ug), HA_GFP (0.08 ug), and FLAG_11E6_WT (2 ug; panel A) or 16E6_WT (2 ug; panel B) into E6AP-null 8B9 cells and HA_NHERF1 expression analyzed by western blot. HA_GFP was co-transfected as a transfection control. Bar graphs below western blots indicate quantitation of HA_NHERF1 protein levels first normalized to HA_GFP to account for transfection variability and then normalized to HA_NHERF1 protein levels in the presence of full length E6AP with no co-expressed E6 (lane 2). E6AP amino terminal truncation to residue 314 displays a lack (with 16E6_WT) or loss (with 11E6_WT) of targeted NHERF1 degradation. The shown experiment is representative of four separate experiments.

We recently reported that both high and low-risk HPV E6 proteins and animal papillomavirus E6 proteins can target the degradation of the signaling adapter molecule NHERF1 in an E6AP-dependent manner [41]. Due to the dependence of high-risk 16E6 on the E6AP 310–320 region for initiating p53 degradation (Fig 3D), we hypothesized that the same E6AP amino terminal region would also be required for both low-risk E6 and high-risk E6 mediated degradation of NHERF1. To test this possibility, we transiently transfected HA_NHERF1, HA_GFP, FLAG_11E6_WT, and various FLAG_E6AP_WT truncations into E6AP-null 8B9 cells. NHERF1 was degraded by 11E6_WT and E6AP truncations until E6AP was truncated to residue 314 (Fig 6A). 16E6_WT was also sensitive to the E6AP 314–875 truncation, as it lost its ability to effectively degrade NHERF1 in the presence of this 314–875 E6AP truncation (Fig 6B). Thus, the same E6AP region that is required for 16E6_L50A to associate with E6AP and for 16E6 to target p53 degradation is also required for degradation of NHERF1 by both low-risk and high-risk E6 proteins.

**Fig 7:**
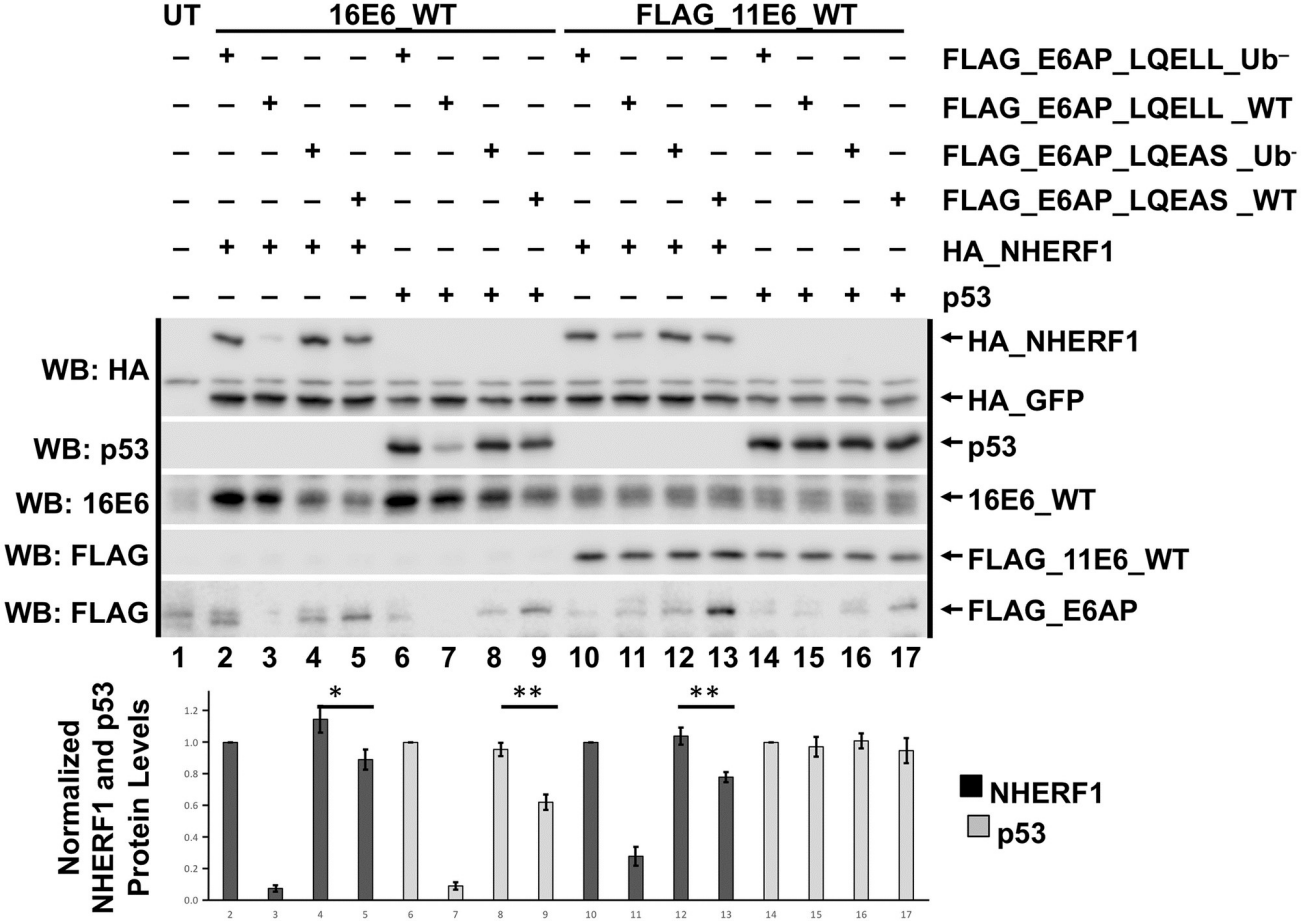
E6AP LQELL is important for efficient degradation of cellular substrates by both high and low-risk E6 proteins. HA_GFP (0.1 ug), human p53 (0.15 ug) or HA_NHERF1 (0.3 ug), and each of the listed FLAG_E6AP variants (0.2 ug) were co-transfected with either untagged 16E6_WT or FLAG_11E6_WT (0.2 ug) in E6AP-null 8B9 cells. E6AP_Ub^–^ indicates a ubiquitin ligase dead E6AP protein that was used as a control. E6AP_LQEAS contains two single amino acid point mutations in the E6 LQELL binding region within E6AP. HA_NHERF1 and p53 protein levels in the presence of the indicated E6 and E6AP proteins were normalized to HA_GFP. The bar graph below the blot represents quantification of either HA_NHERF1 protein levels (black bar) or human p53 protein levels (gray bar) in the presence of the indicated E6 and E6AP proteins. UT = untransfected. Blots from a single representative experiment are shown with quantitation from the average and standard deviation of 4 experimental replicates. *, P< 0.05; ** P<0.01 by Student’s t test.

High-risk 16E6_WT can initiate p53 degradation in the presence of E6AP_WT in which the LQELL motif has been mutated to LQELS [44], even though an association between E6 and E6AP_LQEAS cannot be demonstrated (Fig 1B). To determine whether association of E6 with the E6AP LQELL motif could be further impaired to LQEAS and still enable E6-mediated degradation of cellular substrates, we performed a transient transfection in E6AP-null 8B9 cells with full-length E6AP (either WT or Ub^–^) containing either LQELL or the mutated LQEAS (Fig 7). NHERF1 was degraded in the presence of both 16E6_WT and 11E6_WT when co-expressed with E6AP_LQELL_WT but not E6AP_LQELL_ Ub^–^, reiterating the necessity of E6AP ubiquitin ligase activity for E6-mediated NHERF1 degradation. Although reduced, NHERF1 protein levels when 16E6_WT or 11E6_WT were co-expressed with E6AP_LQEAS_WT did not recapitulate NHERF1 levels with E6AP_LQELL_WT and E6 (Fig 7, compare lanes 5 and 13 to lanes 3 and 11, respectively). But because NHERF1 was slightly degraded by both 16E6 and 11E6 in the presence of E6AP_LQEAS_WT, it appears that very minimal E6 interaction with the E6AP LQELL motif alone activates E6-mediated NHERF1 degradation. These results advance the claim that strong E6-E6AP interactions at the LQELL site are not essential for initiation of E6AP ubiquitin ligase activity [44]. These results were further supported by slight 16E6 degradation of p53 in the presence of E6AP_LQEAS_WT (Fig 7, compare lanes 7 and 9). As expected, 11E6 did not stimulate p53 degradation (lanes 14–17).

#### In vivo competitive binding between LQELL peptides and full-length E6AP for interaction with E6

**Fig 8:**
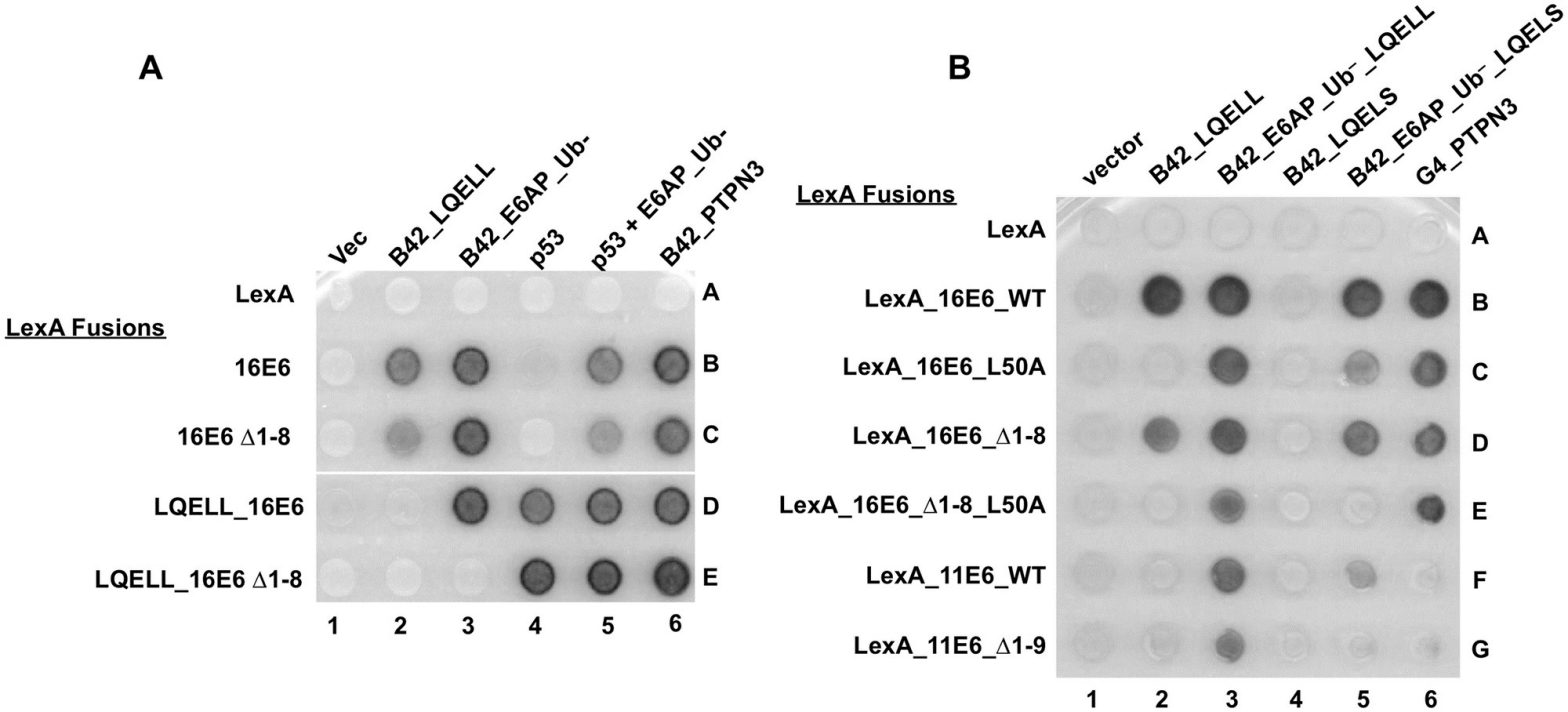
The extreme N-terminus of E6 may play a role in E6AP binding. **(A and B)** Yeast strains expressing the indicated LexA DNA binding domain fusions were mated to prey yeast transfected with B42 transactivator fused E6AP constructs depicted in Fig 1A, transactivator-fused PTPN3, p53, or unfused E6AP_Ub- plus p53 as indicated. LexA_LQELL_16E6 is a triple fusion of LexA to the LQELL peptide of E6AP then to 16E6; in this construction, the LQELL peptide interacts with 16E6 in cis and competes for the binding of B42_LQELL expressed in trans [39]. 16E6_WT was used as a positive control for expression of E6AP proteins, although none of the E6 proteins interacted with B42_LQEAS. The low-risk 11E6 protein is unable to interact with PTPN3 due to a lack of a PDZ binding motif. Deletion of residues 1–8 of 16E6 or 1–9 of 11E6 impairs the Type II interaction of 16E6 with E6AP.

We have previously shown that when LXXLL peptides are fused to the amino-terminus of E6 that those peptides bind to the LXXLL binding site of E6 in the crystal structure [23] and can competitively block the interaction between E6 and its target cellular proteins that are expressed in *trans* [47]. In these experiments, 16E6 shows greater association with full-length E6AP over the isolated E6AP LQELL peptide [39]. However, full-length E6AP is unable to outcompete E6AP LQELL in *cis* with 16E6 that is deleted of the first eight amino acids (16E6_Δ1–8) (Fig 8A, compare spots 3D and 3E). Therefore, we postulated that the first eight amino acids in 16E6 may contribute to the enhanced association of E6 with full-length E6AP compared to the association of 16E6 with the isolated E6AP LQELL motif. 16E6_L50A and 16E6_Δ1–8 each interacted with E6AP_ Ub^–^_LQELS (Fig 8B, spots 5C and 5D). However, the double mutant 16E6_Δ1–8_L50A lost its ability to interact with full-length E6AP_ Ub^–^_LQELS (spot 5E). A multiple sequence alignment indicated that the first eight amino acids of 16E6 were homologous to the first nine amino acids of 11E6. Therefore, we created an 11E6_Δ1–9 mutant to determine whether this amino-terminal E6 region is important in mediating the low-risk E6-E6AP interaction. While 11E6_WT could interact with full-length E6AP_ Ub^–^_LQELS, 11E6_Δ1–9 lost this ability (Fig 8B, compare spots 5F and 5G). Taken together, these data suggest that the initial eight or nine amino acids of 16E6 and 11E6, respectively, play a parallel role in enabling the more robust interaction between E6 and full-length E6AP compared to the interaction of E6 with the isolated LQELL peptide.

### Mapping additional parts of E6AP that contribute to Type II interactions with E6 proteins

**Fig 9:**
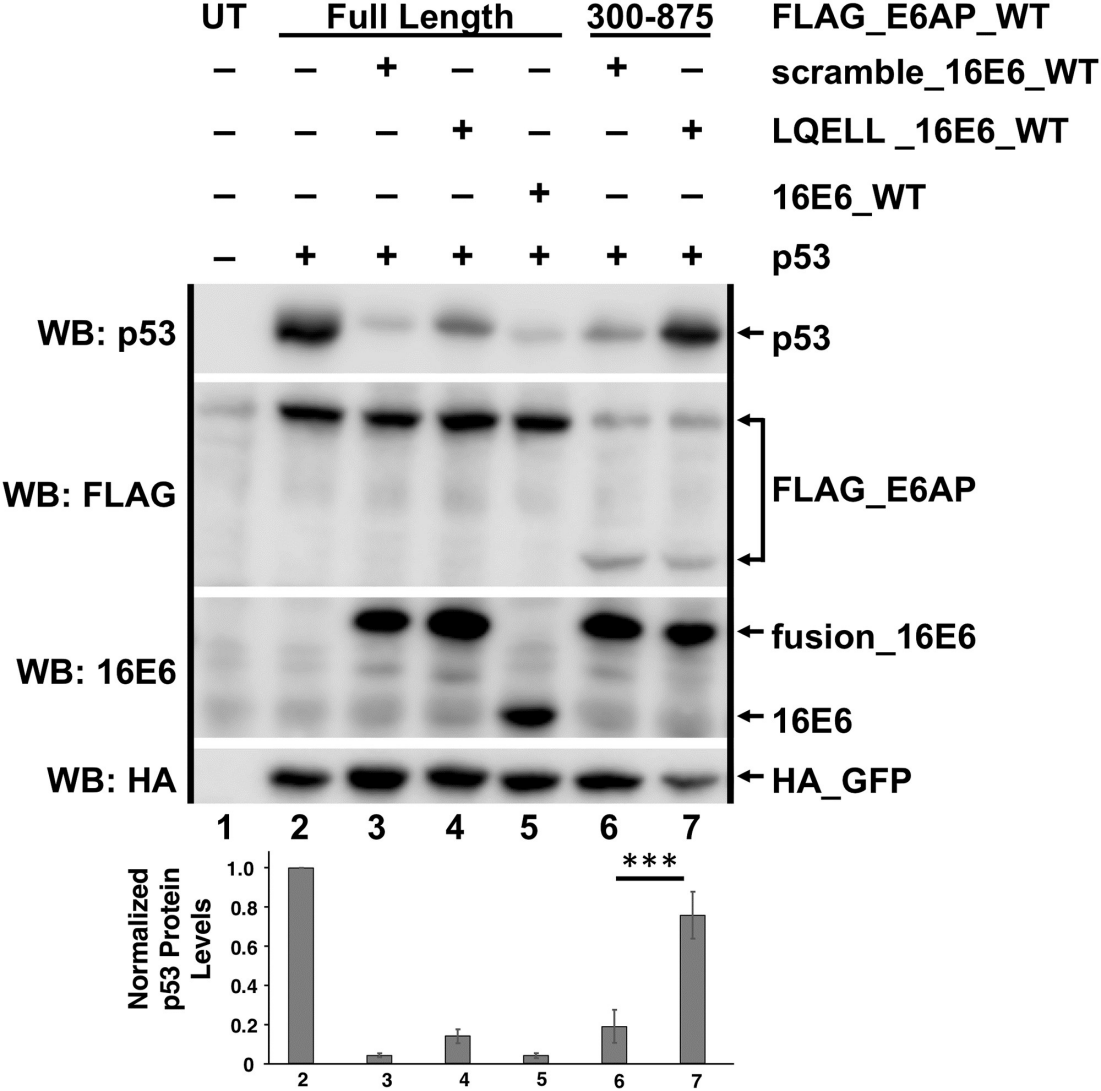
Full-length E6AP outcompetes *cis* LQELL binding to 16E6_WT *in vivo*. Plasmids encoding the indicated FLAG_E6AP_WT (0.5 ug), human p53 (0.5 ug), HA_GFP (0.1 ug), and the listed 16E6 proteins (0.5 ug) were transiently transfected into E6AP-null 8B9 cells and p53 protein expression was analyzed by western blot. LQELL_16E6_WT has an intramolecular interaction such that the 16E6 protein binds to the amino terminally fused LQELL E6AP peptide [39]; scramble_16E6_WT contains a same sized amino terminal fusion with a scrambled LQELL sequence. Unfused 16E6_WT serves as a control to ensure that scramble_16E6_WT degrades p53 as well as untagged 16E6_WT, indicating the amino terminal fusion is not disrupting the fold of the E6 protein. Full-length E6AP outcompeted the LQELL_16E6_WT intramolecular interaction resulting in p53 degradation while the amino terminal E6AP truncation 300–875 did not outcompete the LQELL_16E6_WT interaction. Mean p53 protein levels +/- standard deviations from 4 separate experiments were quantitated and normalized to HA_GFP to account for transfection efficiency. For comparison purposes, the HA_GFP normalized p53 levels were then normalized to p53 protein levels in the presence of full-length E6AP with no co-expressed 16E6 (lane 2). UT = untransfected. Blots from a single representative experiment are shown with quantitation from the average and standard deviation of 4 experimental replicates. *** P<0.001 by Student’s t test.

We have determined that 16E6_L50A which cannot bind the isolated E6AP LQELL peptide but can bind full-length E6AP was able to interact with and initiate p53 degradation in the presence of E6AP 300–875 (Fig 3D). However, both interaction with and degradation of p53 were lost with 16E6_L50A and E6AP truncated past amino acid 315. Therefore, we hypothesized that the 300–315 region of E6AP contributed to enhanced E6 interaction with E6AP *in vivo*. Previous studies have shown that full-length E6AP can outcompete 16E6_WT-LQELL peptide binding in *cis* [39]. To test our hypothesis, we utilized 16E6_WT with an amino terminal fusion of the E6AP LQELL peptide (in *cis*), which was co-transfected with either full-length E6AP_WT or E6AP_WT 300–875 to make an *in vivo* competitive binding experiment between the LQELL peptide in *cis* on E6, and E6AP_WT in trans (Fig 9). The E6AP LQELL peptide in *cis* is sufficient to recruit p53 to 16E6, but is not sufficient for 16E6-mediated degradation of p53 [39]. Therefore, p53 degradation would only be observed if the full-length E6AP or E6AP 300–875 was able to outcompete the LQELL peptide *in vivo* for binding to 16E6. In Fig 9, comparison of p53 levels in lane 4 to lane 7 showed that the LQELL peptide in *cis* of 16E6 more effectively competed against E6AP_300–875 than against E6AP_WT, suggesting that additional regions within the amino-terminus of E6AP upstream of amino acid 300 are important for the observed ability of full-length E6AP to outcompete LQELL binding 16E6 in *cis*.

**Fig 10:**
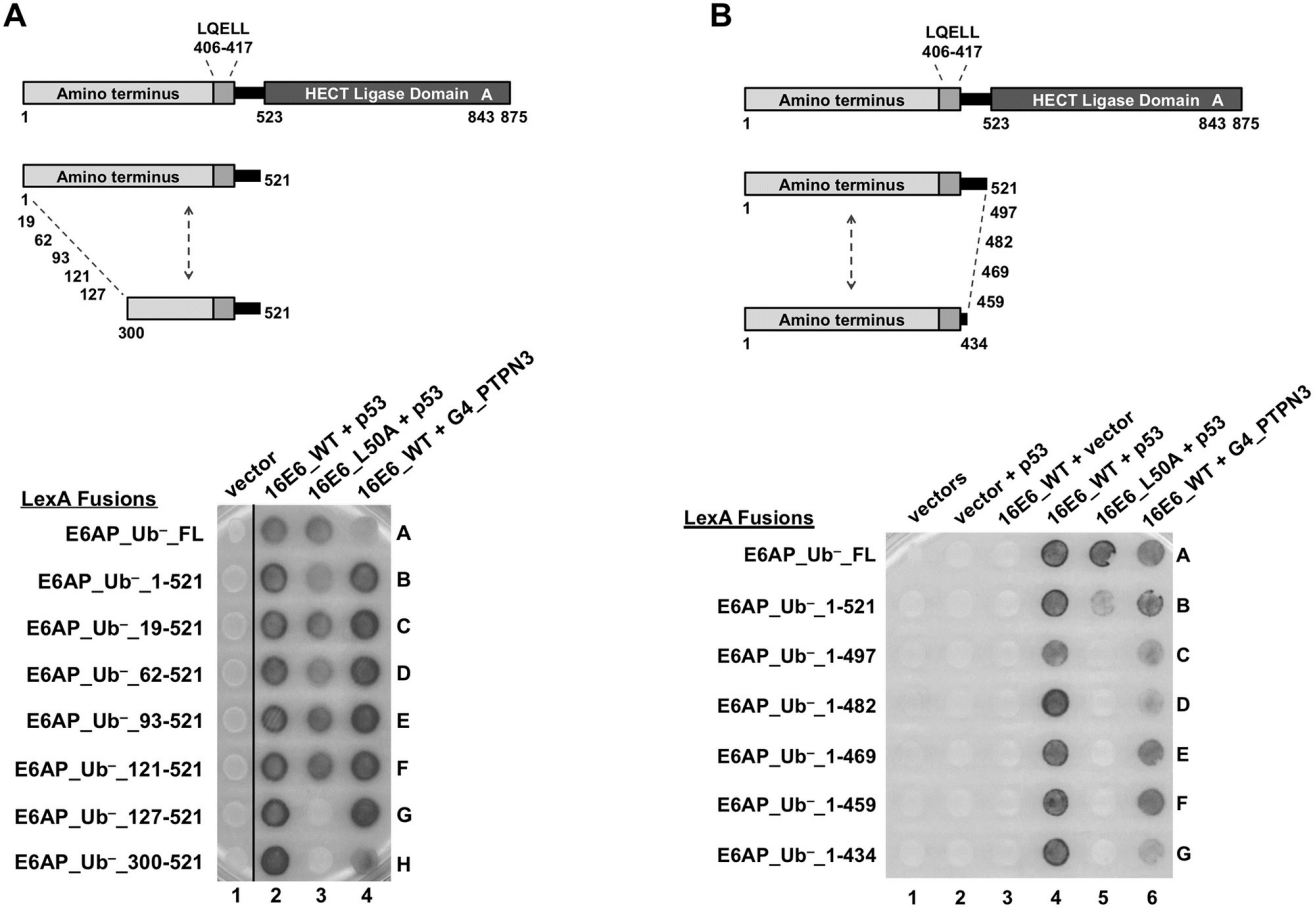
E6AP regions in addition to the LQELL motif in both the amino and carboxy terminus are important in mediating 16E6 binding. **(A)** E6AP residues 121–127 are important for enabling 16E6_L50A interaction and p53 recruitment. At the top, there is a schematic of E6AP and E6AP truncation mutants with yeast hybrid associations shown below. E6AP amino terminal truncations lack the HECT ligase domain, but retain the LQELL E6 binding motif. The indicated yeast expression plasmids were introduced into a LexA-responsive reporter strain by mating. Co-expression of 16E6_WT and p53 with each LexA_E6AP serves as a positive control for E6AP expression and folding. Co-expression of Gal4 (G4) transactivator fused PTPN3 with 16E6_WT and the various LexA_E6AP truncations ensures that 16E6_WT is being expressed and can recruit the PDZ protein PTPN3 to the E6AP protein. Vertical black line indicates removal of irrelevant samples. **(B)** E6AP residues 521–497 are important in mediating 16E6_L50A interaction with E6AP to recruit p53. At the top is a schematic of E6AP and E6AP truncation mutants examined for interaction with 16E6_L50A + p53. E6AP truncations lack the HECT ligase domain but retain the LQELL E6 binding motif. Yeast containing three hybrid plasmids expressing the indicated proteins were mated, selected, and analyzed for interaction as in part A. The PDZ protein PTPN3 co-expressed with 16E6_WT and each E6AP truncation demonstrated ability of 16E6 to bind the E6AP protein and recruit PTPN3.

To identify which region(s) within the E6AP amino terminus upstream of residues 300–315 are important in mediating the E6-E6AP interaction, we constructed amino-terminal E6AP truncation mutants that lacked the HECT domain (Fig 10A). Each truncation retained the E6AP LQELL motif. All of the E6AP truncations enabled 16E6_WT binding and recruitment of p53 (Fig 10A, column 2), which was expected since 16E6_WT can bind to the isolated LQELL peptide and recruit p53. However, in the presence of 16E6_L50A, p53 recruitment was lost when E6AP was amino-terminally truncated from amino acid 121 to 127 (spots 3F and 3G), indicating that the first 127 amino acids of E6AP contribute to 16E6_L50A interaction with E6AP.

We continued to focus on characterizing the region of E6AP amino-terminal to the HECT domain (residues 1–521). Having identified amino acids important in mediating the E6AP-16E6_L50A interaction with 521 as the carboxy-terminal endpoint (Fig 10A), we constructed carboxy-terminal E6AP truncations keeping the amino-terminal endpoint at residue 1 (Fig 10B). In this way, we could determine whether the region of E6AP between the E6AP LQELL motif and the E6AP HECT domain (residue 417–521) contributed to the E6-E6AP interaction. As expected, 16E6_WT successfully recruited p53 to each of the observed E6AP truncations (Fig 10B, column 4), indicating that each E6AP was expressed. However, using 16E6_L50A and carboxy-terminal truncations of E6AP from amino acid 521 to 497, we saw loss of p53 recruitment to the E6AP protein (spots 5B and 5C). This suggests that 16E6 interaction with E6AP *in vivo* is partially mediated by the sequence in E6AP between residues 497 and 521.

**Fig 11:**
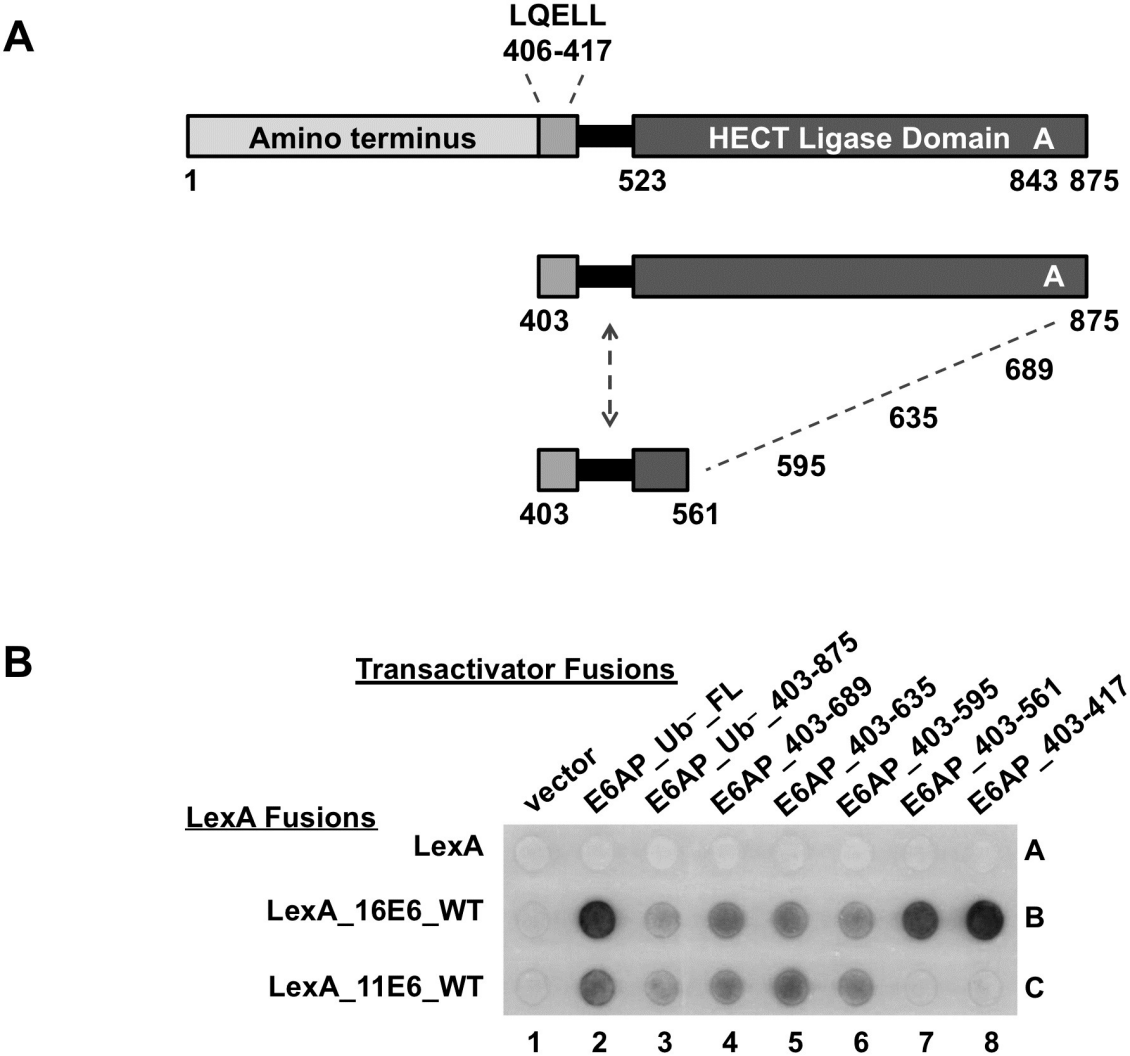
The low-risk 11E6 binding to E6AP requires E6AP residues located in the HECT ligase domain. **(A**) Schematic of full length E6AP and E6AP truncations examined in panel B. Each C-terminal E6AP truncation contains the E6AP LQELL E6 binding motif at the amino terminus. **(B)** Bait yeast transfect with plasmids encoding LexA DNA binding domain fused 16E6_WT, 11E6_WT, or MmPV1E6_WT were mated to yeast containing B42 transactivator fused E6AP truncations as indicated. LexA_16E6_WT served as a positive control for E6AP expression and folding as 16E6 should interact with each E6AP truncation because they all contain the LQELL motif. 11E6 requires E6AP residues 561–595 to interact with E6AP.

Because both low-risk 11E6 and 16E6_L50A are unable to interact with the isolated E6AP LQELL peptide (Figs 1B and 2B or 8B), we speculated that the 11E6 protein would display similar E6AP binding profiles as 16E6_L50A. To determine whether the HECT domain played a role in mediating the E6-E6AP interaction for 11E6, we truncated the entire amino terminal region of E6AP upstream of the E6AP LQELL motif and constructed carboxy-terminal E6AP truncations (Fig 11A). Bait yeast expressing a LexA DNA binding domain fused to either 16E6_WT or 11E6_WT were mated to yeast expressing the various depicted HECT domain E6AP truncations (Fig 11B). Interaction of the E6AP proteins with 16E6_WT indicated each E6AP protein was expressed and folded (row B). While 11E6_WT could interact with E6AP 406–595 (Fig 11B, spot 6C), it lost its ability to interact with E6AP 406–561 (spot 7C), indicating the residues between 561 and 595 in the E6AP HECT domain contribute to the interaction of low-risk 11E6 with E6AP. This was not the case with 16E6_L50A, where interaction with the LQELL motif was not enhanced by the presence of the HECT domain (Fig 3E rows B versus C). E6 fusions from PsPV1, PphPV1, and MmPV1 behaved similarly to HPV11 E6, in that they failed to interact with the isolated LQELL motif (Fig 1B), yet interacted when the HECT domain was present (Fig 3E).

## Discussion

The finding that the 16E6-LQELL peptide interaction alone conferred p53 interaction with E6 [39], followed by the crystallization of p53 bound to the LQELL-16E6 complex [40] represented a tremendous leap forward in understanding papillomavirus-host interactions. However, it has not provided insight into how E6 interacts with the full-length E6AP protein, the significance of E6AP regions outside of the LQELL peptide in mediating E6-E6AP interaction, or E6 stimulation of the ubiquitin ligase activity of E6AP. Here, we show that E6AP-binding E6 proteins from both human and non-human papillomaviruses require regions of E6AP in addition to LQELL, and furthermore that these regions can differ among the various E6 proteins tested.

**Fig 12:**
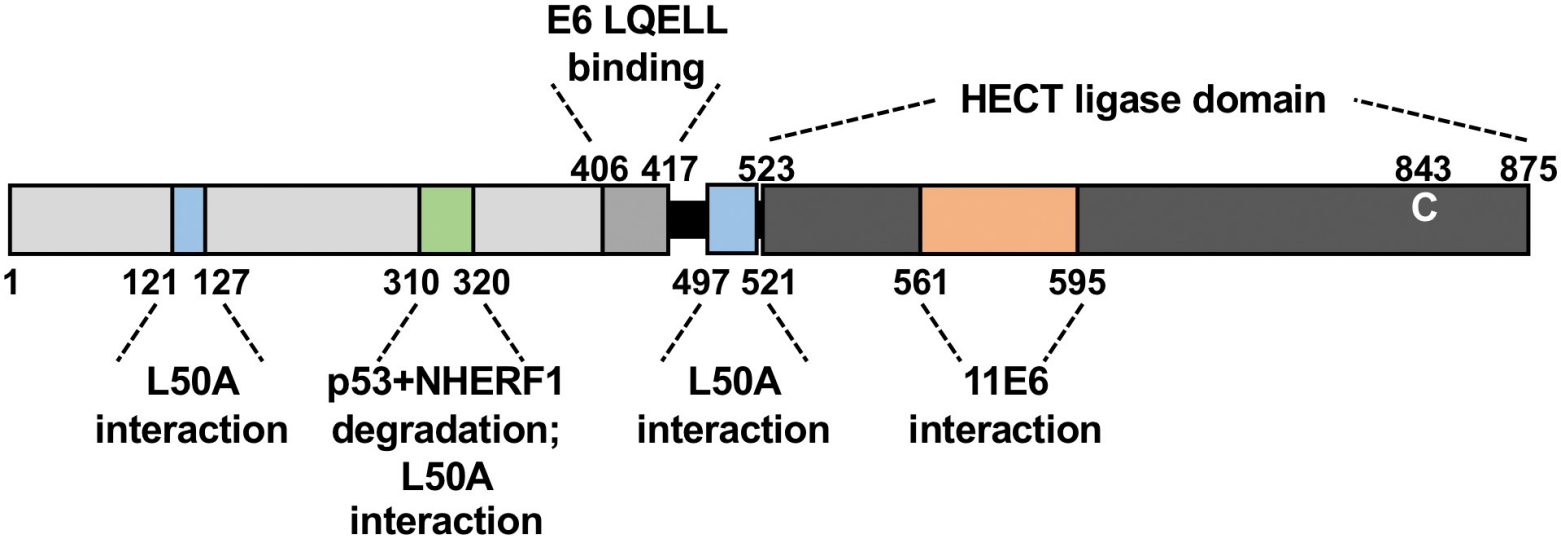
Summary schematic: Identified regions in E6AP important in mediating E6 binding and cellular protein degradation. The herein identified and described regions within E6AP are indicated as being important in mediating E6 binding and/or in E6-mediated degradation of cellular proteins.

Our use of the 16E6_L50A mutant led to the identification of three regions of E6AP that contribute to the association of 16E6 with E6AP, illustrated in Fig 12: two in the N-terminus (amino terminal to the LQELL motif; Figs 3 and 10A) and one carboxy-terminal to the E6 LQELL binding motif (Fig 10B).

The first amino-terminal region is ablated by deletion of the first 127 amino acids of E6AP and contributed to the association of 16E6_L50A with E6AP, but was not required for E6-stimulation of E6AP ubiquitin ligase activity. The second, ablated by deletion from E6AP residues 310–320, augments the interaction of 16E6_L50A with E6AP and is required for initiation of E6AP ubiquitin ligase activity (Fig 3) for both high and low-risk E6 to induce degradation of cellular NHERF1 (Fig 6). Finally, carboxy to the E6 LQELL binding motif, a region (497–521) just upstream of the HECT domain (525–875), augments the ability of 16E6_L50A to interact with E6AP lacking the HECT domain (Fig 10B).

Interestingly, we found that unlike high-risk 16E6, low-risk 11E6, which does not interact with the isolated LQELL peptide, requires a region (residues 561–595) located within the HECT domain. (Fig 11B), as did animal papillomavirus E6 proteins (Fig 3E). Interestingly, 16E6_L50A is unable to interact with E6AP when the amino terminus is deleted (Fig 3B).

The ability to bind the isolated E6AP LQELL peptide *in vivo* was first discovered through study of high-risk 16E6 and Bovine Papillomavirus type 1 E6 [21, 22, 37, 39], but this is not a high-risk trait. The rhesus monkey MmPV1 is a high-risk virus [48], its E6 protein targets the degradation of p53 [27], and we did not detect its interaction with E6AP LQELL peptide alone (Fig 1B, spot 2N). Additionally, we found E6 proteins that are from viruses that are predominately cutaneous (SsPV1, HPV7, HPV10) as well as E6 proteins that are derived from predominately mucosal viruses (HPV16 and UmPV1) can bind the isolated E6AP _LQELL peptide.

Through our screen (Fig 1B) we propose two distinct types of E6-E6AP interactions: Type I and Type II. In Type I, the E6 protein can bind the isolated E6AP LQELL peptide (as in 16E6). In Type II, the E6 protein cannot bind isolated E6AP LQELL (as in 11E6) but binds full-length E6AP. In our study, while all E6 proteins interacted with E6AP_Ub- containing the wild-type LQELL motif, some did not interact when the LQELL motif was mutated to LQELS, and none interacted with E6AP when the LQELL motif was mutated to LQEAS. This indicates that whatever the mechanism of auxiliary regions of E6AP in promoting E6 association with E6AP, interaction requires the interaction of E6 with the LQELL motif.

The interaction of 11E6 with E6AP was lost upon deletion of E6AP from amino acid 595 to 561 in the HECT domain (Fig 11B). In contrast, 16E6_L50A did not interact with E6AP_331–875_UB-, which encompasses the E6AP_403–875_Ub- fragment (Fig 3B). It is remarkable that these two E6 proteins do not share the same interaction profile with E6AP mutants given neither 11E6 nor 16E6_L50A interact with isolated E6AP LQELL peptide, and suggests evolutionary drift in the role of auxiliary regions of E6AP in contributing to the E6-LQELL interaction. It is possible that as E6 proteins diverged to target different intracellular targets, the particulars of their associations with E6AP also diverged. It is interesting that E6 proteins from HPV11, PsPV1, PhPV1, and MmPV1 all fail to interact with the isolated LQELL peptide but gain Type II E6AP interaction through additional E6AP sequences.

E6 is a potent activator of the E3 function of E6AP [49] but the mechanism behind that activation remains unclear. E6AP residues 310–320 (located amino-terminal to the E6AP LQELL motif) are required for 16E6_L50A to associate with E6AP in yeast and for recruitment of p53, and the same region is required for the degradation of p53 and NHERF1 in mammalian cells and the co-immune precipitation of E6 and E6AP from mammalian cells (Figs 3B–3E, 4 and 6). There is thus a correlation between forming a Type II interaction with E6AP (illuminated by 16E6_L50A interaction with E6AP) and 16E6 stimulation of E6AP E3 ubiquitin ligase activity. Our Y3H results are corroborated in E6AP-null murine 8B9 cells, the use of which enabled us to measure both p53 and 16E6 protein levels (Fig 3D). Because E6 protein is stabilized by E6AP [46] we initially hypothesized that the stability of 16E6 would directly relate to its ability to initiate p53 degradation. However, quantification of both p53 and 16E6 levels in the presence of various E6AP truncations clearly indicated that 16E6 does not have to be stabilized by E6AP in order to target the degradation of p53 (Fig 3D). Indeed, 16E6_L50A displays a loss of protein stability (mimicking E6 protein levels when there is no E6AP present) in the presence of E6AP 310–875, but p53 is still being degraded (Fig 3E). Additionally, 16E6_WT protein levels indicate loss of E6 stability with E6AP 315–875, yet p53 is still degraded. We attempted to identify a single amino acid within 310–320 in the context of full-length E6AP required for mediation of E6-induced p53 degradation (Fig 5); no single point mutant recapitulates our 310–320 truncation data. Soft mutations (e.g. mutation to alanine) may be insufficient to disrupt our desired phenotype and strong mutations could illuminate a single, necessary residue.

Because 16E6_WT is able to initiate p53 degradation in the presence of E6AP 315–875, while 16E6_L50A requires E6AP 310–875, we speculate that the capacity of 16E6_WT to interact with LQELL above some critical threshold aids in stimulation of E6AP E3 ligase activity. This is illustrated by the absence of detectable association of E6 proteins in vivo with B42_E6AP_LQEAS_Ub- (Fig 1B) compared to the small residual activity of E6AP_LQEAS against p53 plus E6 (Fig 7). Indeed, 11E6, which is unable to bind the isolated E6AP LQELL peptide, stimulates E6AP ligase activity to target NHERF1 for degradation, but also requires E6AP residues 310–320 (Fig 6). A recent study utilized chemical cross-linking coupled to mass spectrometry (XL-MS) to model the E6-E6AP-p53 trimeric complex and postulated that the interaction results in both E6 and p53 being in close proximity to the E6AP active cysteine residue in the HECT domain [50]. It is possible that the proposed conformational orientation of E6-E6AP-p53 requires E6AP amino acids 310–320 and that orienting E6 and the cellular substrate close to the E6AP HECT domain activates E6AP ubiquitin ligase activity. Furthermore, previous studies indicate that E6AP-mediated ubiquitin chain formation relies on the N-terminal region of E6AP [49, 50]. If this specifically applies to E6AP residues 310–320, it may be that the processivity of ubiquitin chain formation mediated by E6AP is disrupted with the E6AP 320–875 truncation.

Evidence suggests that 16E6 displays a higher association with full-length E6AP over the isolated LQELL peptide [39, 51, 52]. We reasoned that because E6AP region 310–320 is important for enhanced association of 16E6_L50A, it may also be required for the observed preferred association of E6 for full-length E6AP over LQELL. Surprisingly, we found that the ability of full-length E6AP to outcompete E6 binding to the E6AP LQELL peptide is lost when E6AP is truncated to residue 300 (Fig 9). This suggests that regions in E6AP between residue 1 and 300 must be important in full-length E6AP outcompeting the LQELL peptide for E6 binding, although these regions are disposable for 16E6_L50A binding in the context of full-length E6AP and for E6 initiation of E6AP ubiquitin ligase activity. Utilizing amino-terminal truncations of E6AP that were deleted of the HECT domain, we found deletion from E6AP residues 121–127 ablated 16E6_L50A interaction with E6AP. The E6AP 121–127 region may be required to enable full-length E6AP to outcompete the LQELL peptide in *cis* with 16E6. As stated previously, XL-MS based modeling of the E6-E6AP interaction proposed that E6AP undergoes a conformational rearrangement upon binding to E6, and that the N and C-terminal regions of E6AP are brought into close proximity [50]. This E6AP conformational rearrangement depends on E6AP residues amino-terminal to amino acids 300. We hypothesize that this conformational rearrangement of E6AP is important for higher association with E6 and as a result, E6AP 300–875 (deleted of its N-terminus) is unable to outcompete LQELL for binding to 16E6_WT. It is possible that E6AP residues 121–127 are required for the E6AP conformational rearrangement and that E6AP 121–875 would be able to outcompete LQELL bound in *cis* to 16E6. Further testing is warranted to further our understanding of this relationship.

To better understand the E6-E6AP interaction we strove to characterize regions within E6 as well as E6AP. E6 proteins are generally small (around 150 amino acids) and so truncations risk protein misfolding. However, 16E6 deleted of its first eight amino acids (16E6_Δ1–8) can still bind full-length E6AP, stimulate degradation of E6AP in an overexpression system, and target p53 for degradation [44]. We noticed that full-length E6AP cannot outcompete LQELL bound in *cis* to 16E6_Δ1–8 (Fig 8A), suggesting that the first eight residues of 16E6 are important in mediating *in vivo* higher association (Type II) of full-length E6AP to the E6 protein. We hypothesized that there are two main E6-E6AP interaction mechanisms: one which has been described, characterized, and crystalized between the E6AP LQELL motif and E6 (see [23]) and one which requires E6AP auxiliary regions and the first eight amino acids of 16E6. We found that, like 16E6_WT, 16E6_Δ1–8 interacts with isolated E6AP_LQELL peptide, peptide, full-length E6AP_LQELL (Fig 8A, row B vs row C), and full-length E6AP_LQELS (Fig 8B, spot 5B vs 5D). Therefore, the deletion of residues 1–8 in 16E6 is not sufficient to prevent interaction with E6AP auxiliary regions and inhibit binding to E6AP_LQELS (a mutant which should minimize the E6-E6AP_LQELL interaction; Fig 8B, spot 5D). This observation led us to adjust our hypothesis: there is a dual modality of E6-E6AP interaction that is mediated from both the E6 and E6AP perspective; such that mutating one of the proteins (e.g. E6AP_LQELS) does not completely attenuate the corresponding interaction (e.g. E6-E6AP via LQELS). Indeed, the 16E6_Δ1–8_L50A double mutant retains ability to interact with full-length E6AP_LQELL, but can no longer interact with full-length E6AP_LQELS (Fig 8B, spot 3E vs. 5E). Further support of our hypothesis stems from examining low-risk 11E6 (Fig 8B, rows F and G), which like 16E6_L50A, cannot bind the LQELL peptide alone. 11E6Δ1–9 (homologous to 16E6Δ1–8) could not interact with full-length E6AP_LQELS, but did interact with full-length E6AP_LQELL (spot 3G vs 5G).

Here we show that divergent E6AP-binding E6 proteins require different auxiliary binding regions within E6AP in order to form the E6-E6AP complex, identifying previously undescribed regions within E6AP in both the amino and carboxy-termini domains that are important in enabling the E6-E6AP interaction (Fig 12). We also describe a region within the amino terminus of E6AP that is required for both high and low-risk E6 proteins to stimulate E6AP ubiquitin ligase activity (Fig 12). While our approach does not preclude E6 interactions with E6AP regions outside of those we have identified as important, it does highlight regions of E6AP that both 16E6_L50A and 11E6 require to maintain the important interaction with E6AP. Taken together, these *in vivo* data will inform future structural studies of this important heterodimer.

## Materials and methods

### Cells and cell culture

E6AP-null 8B9 cells (a gift of Dr. Lawrence banks, ICGEB, Italy) [53] were maintained and transfected using polyethylenimine (PEI) as previously described [39].

### Transfections

Epitope tagged E6AP, GFP, E6, and NHERF1, as well as native p53 were all transiently expressed from the pcDNA3 plasmid. HA-tagged human NHERF1 originated from Vijaya Ramesh’s laboratory (from Addgene, plasmid 11635). 16E6 point mutants were created using QuikChange primer design (Agilent Technologies). 16E6ΔPBM was created by mutating the PBM of 16E6 from ETQL* to EL* and E6AP_Ub^–^ was created by mutating the active cysteine residue at position 843 to an alanine (C843A). E6AP numbering based on E6AP isoform II. The E6AP constructs utilized express human E6AP isoform III. NHERF1 truncations were PCR generated and sequenced.

### Antibodies and Western blots

12 well plates of transfected murine 8B9 cells were lysed in 0.5X IGEPAL lysis buffer as described previously [27]. All lysates were resolved by SDS-PAGE electrophoresis and transferred to PVDF membranes. Antibodies: rabbit anti-HA (Bethyl Laboratories, Inc.), mouse anti-HA (MAb clone 12CA5), rabbit anti-FLAG M2 (Sigma), mouse anti-p53 (MAb clone Ab-8, ThermoFisher Scientific), mouse anti-FLAG epitope (clone M2, Sigma) and anti-16E6 MAb 6G6 (a generous gift from Arbor Vita Corporation). Detection of blot signals was captured and analyzed using a Syngene G:Box (Syngene USA, Frederick, Maryland).

### Yeast hybrid expression

Yeast LexA-responsive reporter strain YSV1280E is derived from the yeast 2-hybrid reporter strain EGY48 [54] and is (MAT-alpha, *ade2*, *his3*, *trp1*, *ura3-52*, *leu2*::*pLEU2-lexAop6)* and contains a LexA responsive lacZ 2-micron reporter plasmid with HIS3 selection. YPH499 [55] is mat-a, *ura 3–52*, *ly2-801*, *ade2-101*, *trp1-Δ63*, *his3-Δ200*, *leu2Δ1* and was used to introduce plasmids by mating into YSV1280E strains. Empty expression plasmids containing appropriate selection markers were introduced into mating strains so that for any particular experiment all selected yeast contained the same number of plasmid types and expressed the same auxotrophic markers. Yeast mating, selection and transfection were as previously described [22]. LexA fusions were expressed from a yeast 2-micron plasmid which expresses LexA fusions from a galactose-regulated promoter and is selected with URA3 expression in yeast and chloramphenicol in bacteria. P53 and PTPN3 expression is from a galactose-regulated yeast 2-micron expression plasmid with tryptophan selection in yeast and ampicillin selection in bacteria. Native E6 expression was from a yeast 2-micron galactose-regulated expression plasmid with ade2 selection in yeast. For yeast 2-hybrid experiments, interacting proteins were fused to either the B42 transactivator or to GAL4 transactivator as indicated in the figure; p53 expression was native p53.

## Supporting information

S1 Fig 16E6_L50A and R102A mutants have similar interaction traits with E6AP deletion mutants. The indicated LexA_E6AP fusions are expressed in rows, while the indicated 16E6 mutants together with p53 in columns in a yeast 3-hybrid assay. Both 16E6_L50A and 16E6_R102A have reduced interaction with isolated E6AP LQELL peptide compared to 16E6_WT.
(TIF)
