# Supplementary figures and images for: Multiple regions of E6AP (UBE3A) contribute to interaction with papillomavirus E6 proteins and the activation of ubiquitin ligase activity
Source: PLoS Pathog. 2020 Jan 23;16(1):e1008295. doi: 10.1371/journal.ppat.1008295 (PMC6999913; doi:10.1371/journal.ppat.1008295)

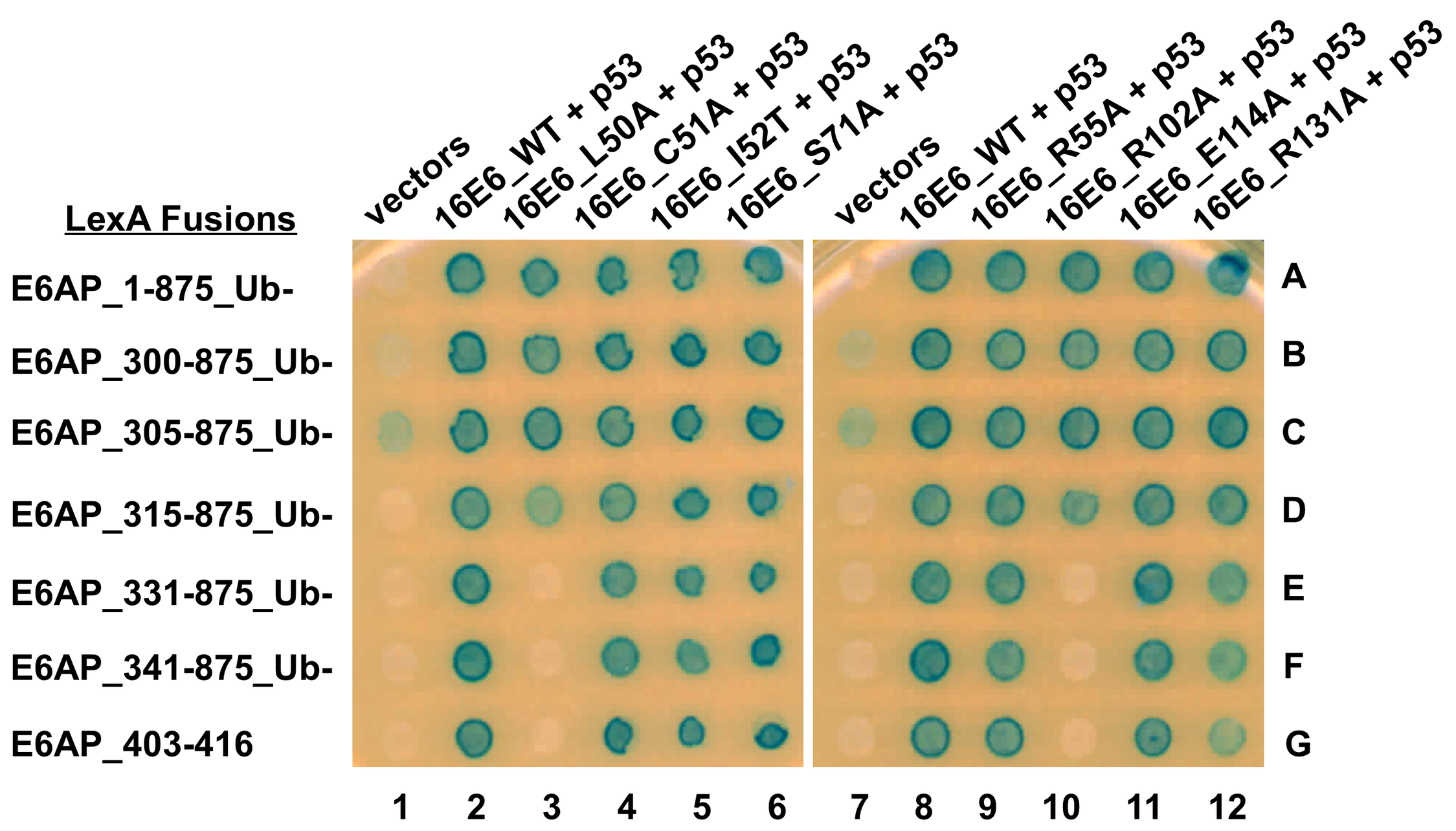

Supplement: S1 Fig — The indicated LexA_E6AP fusions are expressed in rows, while the indicated 16E6 mutants together with p53 in columns in a yeast 3-hybrid assay. Both 16E6_L50A and 16E6_R102A have reduced interaction with isolated E6AP LQELL peptide compared to 16E6_WT. (TIF) [file ppat.1008295.s001.tif]
